# Supplementary material for: Laminin Levels Regulate Tissue Migration and Anterior-Posterior Polarity during Egg Morphogenesis in Drosophila
Source: Cell Rep. 2017 Jul 5;20(1):211–23. doi: 10.1016/j.celrep.2017.06.031 (PMC5507772; doi:10.1016/j.celrep.2017.06.031)
Supplement: Document S1. Supplemental Experimental Procedures, Figures S1–S5, and Tables S1–S3 [file mmc1.pdf]

Cell Reports, Volume *20*

Supplemental Information

Laminin Levels Regulate Tissue Migration and Anterior-Posterior Polarity during Egg Morphogenesis in *Drosophila*

María C. Díaz de la Loza, Alfonsa Díaz-Torres, Federico Zurita, Alicia E. Rosales-Nieves, Emad Moeendarbary, Kristian Franze, María D. Martín-Bermudo, and Acaimo González-Reyes

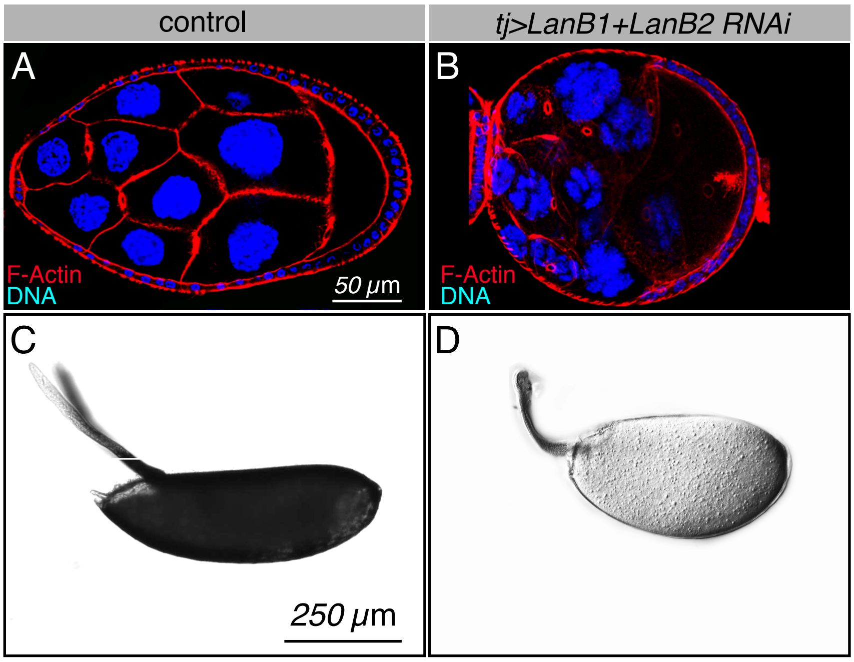

Supplemental Figure 1

Supplemental Figure 1 (related to Figures 1 and 2): Strong laminin depletion blocks egg elongation and follicle migration. **(A)** S5-S7 wild-type egg chambers are elongated along the anterior-posterior axis. **(B)** Strong depletion of laminin levels in *tj>LanB1+LanB2 RNAi* ovaries prevents egg elongation. Thus, S5-S7 egg chambers appear rounded (100% of cases, n=54). **(C)** The large majority of eggs laid by control females are properly elongated (number of eggs analysed (n)=178; length/width ratio ~2.75). **(D)** *tj>LanB1+LanB2 RNAi* females lay 86% eggs rounder than controls (n=89; length/width ratio <2.5). Filamentous actin was detected with rhodamine-labelled phalloidin.

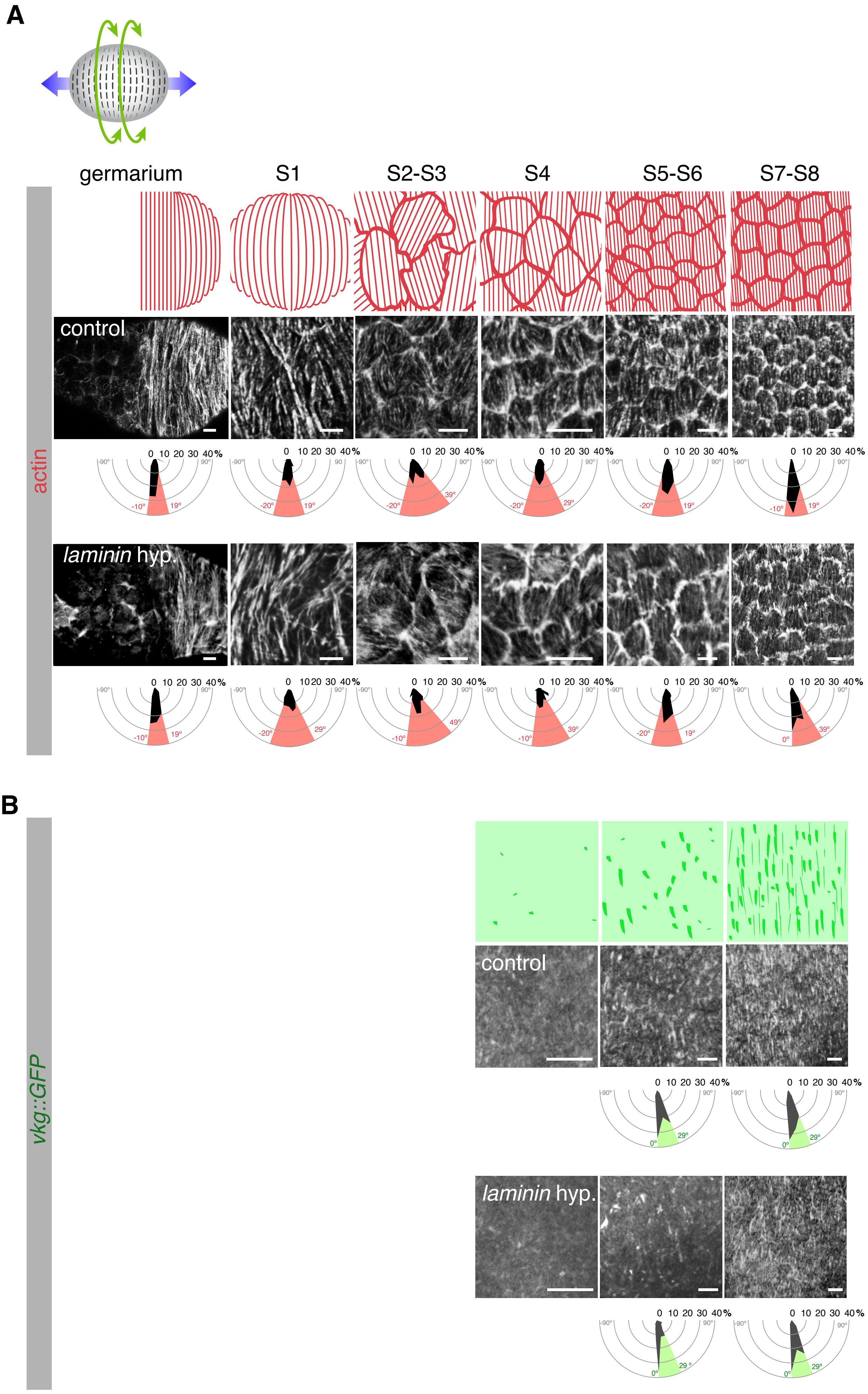

Supplemental Figure 2

Supplemental Figure 2 (related to Figure 2): Basal planar cell polarity of follicle cells and organisation of Col IV fibrils is not affected in *laminin* hypomorphic ovarioles. (A) Schematic representation of the distribution of basal actin filaments in follicle cells during egg chamber development (top). Visualisation of filamentous actin with rhodamine-labelled phalloidin in control (middle) and *laminin* hypomorphic ovarioles (bottom). Actin filaments are clearly organised perpendicular to the anterior-posterior axis of the ovariole in germarial stages and from S5 to S8. S2-S4 egg chambers display a less obvious polarisation. Radial diagrams indicate the percentage of bundles found within a given sector. The black areas correspond to the data reported in Table S2. Angles delimiting the sectors (depicted in pink) are shown. **(B)** Schematic representation of the organisation of the basement membrane component Col IV (top). Immunodetection of the protein trap Vkg::GFP in control (middle) and *laminin* hypomorphic ovarioles (bottom) show that Collagen IV is polarised perpendicular to the ovariole's AP axis from S5. Radial diagrams indicate the percentage of fibrils found within a given sector. The black areas correspond to the data reported in Table S4. Scale bars = 5 microns.

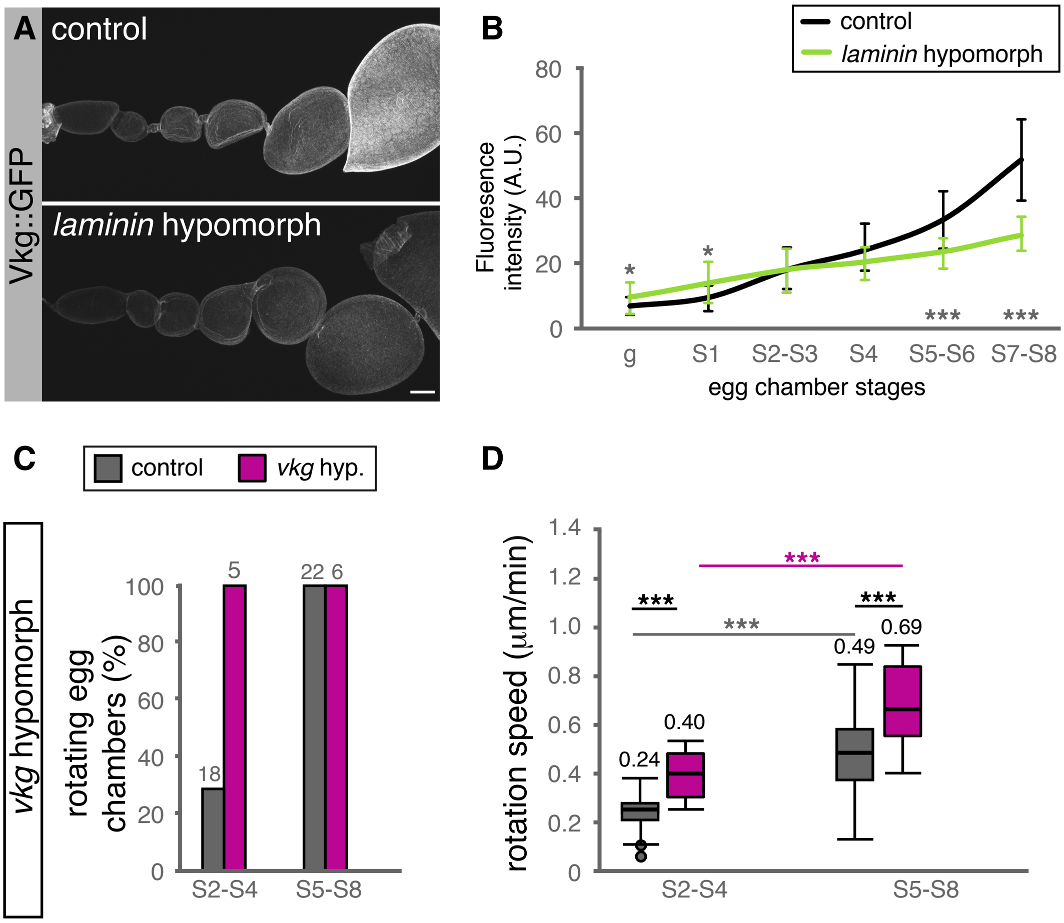

Supplemental Figure 3

Supplemental Figure 3 (related to Figure 2): Col IV deposition is affected in S7-8 *laminin* hypomorphs. Col IV is required for proper rotation. **(A)** Immunodetection of Col IV by means of the protein trap Vkg::GFP in control and *laminin* hypomorphic ovarioles using an anti-GFP antibody. Image is a maximum projection of at least 15 sections along the Z-axis. **(B)** Quantification of the immunofluorescence signal in different egg chambers is shown. Mean and standard deviation of measurements in 3 to 5 ovarioles are indicated. **(C)** Onset of rotation and **(D)** quantification of rotation speed of *vkg* hypomorphic follicles at different stages. Horizontal lines in boxes represent MEDIAN values of a minimum of 11 and a maximum of 103 measurements per genotype and stage. Values above boxes indicate MEAN rotation speeds. See Movies S1 and S7. P values of two-tailed t-tests <0.05 were considered statistically significant (*: $P<0.05$, **: $P<0.005$, ***: $P<0.001$). Scale bar = 25 microns.

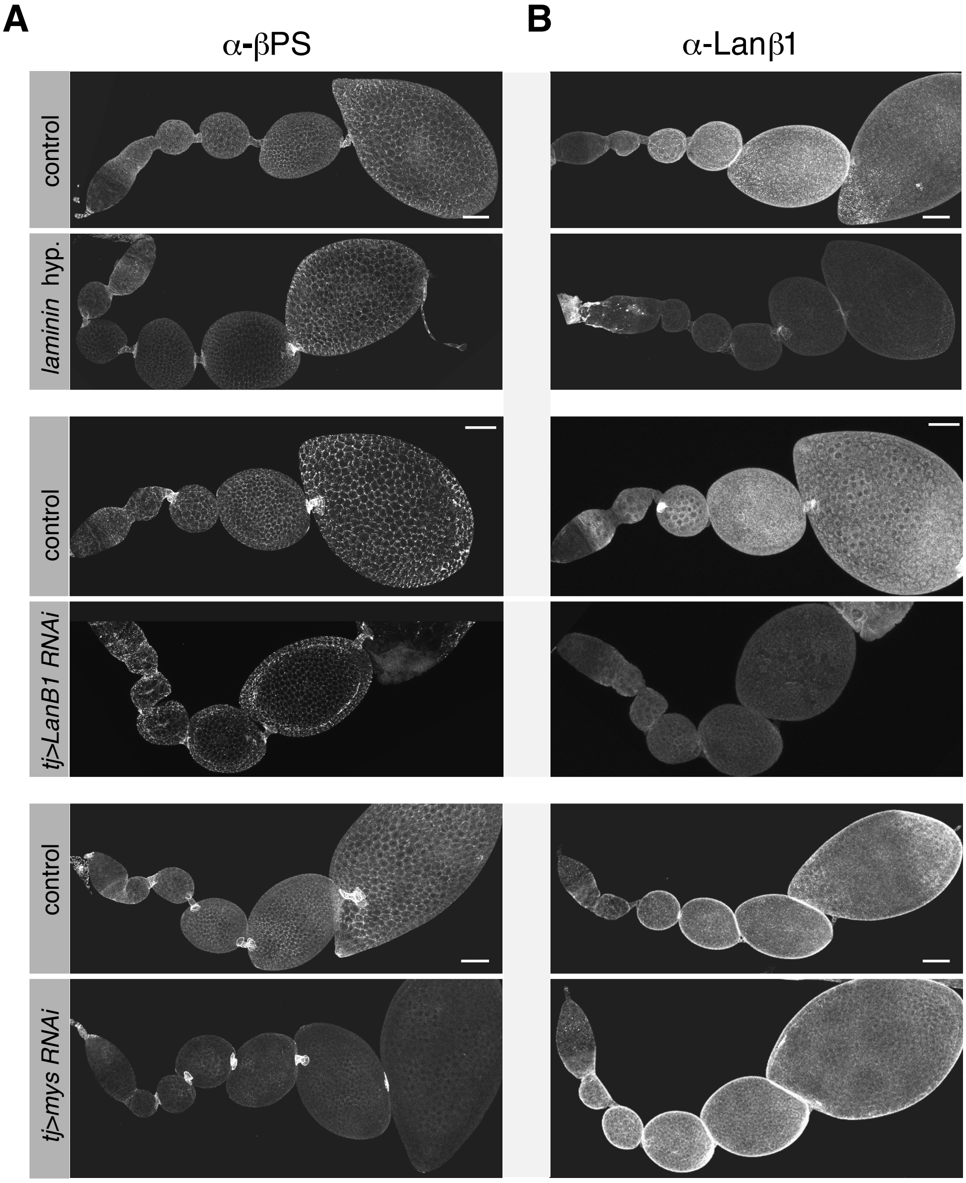

Supplemental Figure 4

Supplemental Figure 4 (related to Figure 4): **Visualisation of ßPS integrin and Laminin ß1 in control and experimental ovarioles.** **(A)** Immunodetection of ßPS and **(B)** Laminin ß1 during oogenesis in controls and in different genetic backgrounds. See Supplementary Table 1 for the quantification of fluorescence data. Images are maximum projections of at least 15 sections along the Z axis. Scale bars = 25 microns.

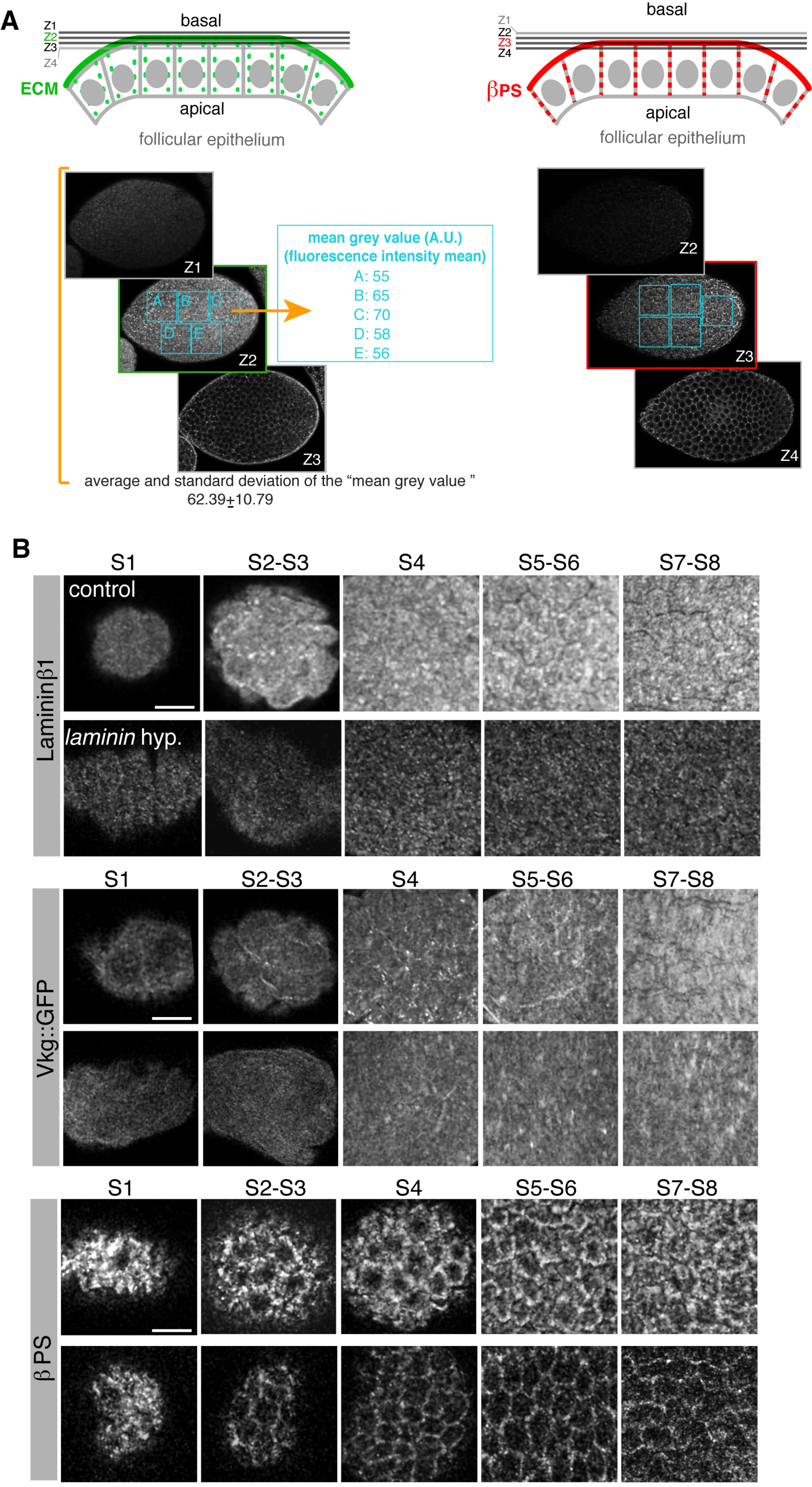

Supplemental Figure 5

Supplemental Figure 5 (related to Figures 1, 4, S3 and S4): **Quantification of immunofluorescence of basement membrane components and basal integrin**. **(A)** Schematic representation of the focal planes used to quantify immunofluorescence levels of BM components (green, left) and basal integrin (red, right). Confocal sections were taken through the egg chamber at 0.8-1.0 μm intervals (Z1, Z2, Z3 and Z4). Z2 cut through the plane of the BM (green line; to quantify ECM components). Membrane or cytoplasmic signals (green dashed lines) were discarded in BM measurements. Z3 focused on the basal side of the follicle cells (red line; to quantify integrin levels). Fluorescent signal from the lateral membrane of follicle cells (red dashed lines) was avoided in integrin measurements. For a given confocal section, five non-overlaping flat areas (cyan squares) were chosen to avoid edge effects and their mean "grey value" was calculated for each area using Imaris and/or ImageJ software (as shown for the BM example in **A**; see Suppl. Experimental Procedures for further details). Average fluorescence intensities ± standard deviations were calculated from ≥3 egg chambers of the appropriate stage (i. e., at least 15 measurements per stage). **(B)** Examples of high magnification images of basal laminin, Col IV and integrin immunofluorescence used for the analyses of protein levels in both control and experimental S1-S8 egg chambers. Scale bar = 5 microns.

SUPPLEMENTAL TABLE 1 (related to Figs. 1, 2, 4, S3 and S4)

Quantification of the immunofluorescence signal (A.U.) of Vkg::GFP, α-βPS and α-Laminin β1, and of rotation speed during oogenesis

genotype	egg chamber stage					
	**germarium**	**S1**	**S2-S3**	**S4**	**S5-S6**	**S7-S8**
**collagen IV levels**						
control	**6.10**±2.41	**9.15**±4.46	**17.91**±7.00	**24.05**±7.07	**33.34**±8.82	**52.00**±13.02
laminin hypomorph	**8.86**±4.72	**13.77**±6.19	**18.12**±6.29	**20.00**±5.59	**22.90**±4.70	**28.50**±5.26
fold change	*1.45*	*1.50*	*1.01*	*0.83*	*0.67*	*0.55*
	*	*			***	***
**laminin levels**						
control	**21.66**±3.63	**41.01**±15.3	**48.78**±12.7	**67.91**±5.84	**67.42**±21.86	**62.39**±10.79
laminin hypomorph	**22.82**±3.12	**21.53**±4.31	**19.51**±1.63	**20.58**±3.66	**17.66**±2.13	**16.60**±2.80
fold change	*1.05*	*0.53*	*0.40*	*0.31*	*0.26*	*0.27*
		***	***	***	***	***
control	**21.81**±3.63	**40.42**±13.6	**46.53**±11.3	**59.78**±15.31	**66.98**±18.74	**60.57**±13.40
tj>LanB1 RNAi	**13.65**±4.32	**21.82**±7.62	**31.03**±4.00	**28.31**±9.06	**27.54**±9.18	**28.76**±8.02
fold change	*0.63*	*0.54*	*0.67*	*0.47*	*0.41*	*0.47*
	***	***	***	***	***	***
control	**27.20**±2.05	**43.29**±9.58	**46.01**±11	**53.83**±5.65	**61.55**±9.09	**57.42**±15.42
tj>mys RNAi	**28.21**±4.48	**69.49**±7.81	**73.54**±12.7	**74.04**±8.33	**67.62**±14.91	**56.08**±16.67
fold change	*1.04*	*1.61*	*1.60*	*1.38*	*1.10*	*0.98*
		***	**	***		
**integrin levels**						
control	**12.00**±5.42	**42.24**±4.40	**36.93**±2.26	**34.79**±3.52	**38.15**±4.02	**38.32**±5.16
laminin hypomorph	**12.20**±2.86	**30.77**±5.49	**18.75**±5.17	**14.77**±4.77	**18.88**±4.19	**21.30**±5.70
fold change	*1.02*	*0.73*	*0.51*	*0.42*	*0.49*	*0.56*
		***	***	***	***	***
control	**14.53**±6.27	**44.44**±13.1	**36.86**±9.98	**38.55**±8.42	**39.85**±6.57	**39.39**±7.52
tj>LanB1 RNAi	**13.71**±3.91	**26.48**±10.9	**26.76**±8.77	**27.98**±9.33	**28.34**±8.21	**27.95**±3.78
fold change	*0.69*	*0.60*	*0.73*	*0.76*	*0.78*	*0.78*
		***	***	***	***	***
control	**19.20**±3.11	**43.10**±8.31	**34.54**±5.67	**34.22**±4.77	**38.06**±5.09	**38.20**±9.63
tj>mys RNAi	**20.72**±5.16	**12.90**±2.96	**18.41**±4.30	**22.18**±3.81	**24.02**±5.33	**19.88**±2.45
fold change	*1.08*	*0.30*	*0.53*	*0.65*	*0.63*	*0.52*
		***	***	***	***	***
**Rotation speed (µm/min)**			**S2-S4**		**S5-S8**	
control			**0.24**±0.08		**0.49**±0.17	
laminin hypomorph			**0.56**±0.18		**0.62**±0.27	
tj>LanB1 RNAi			**0.50**±0.09		**0.79**±0.16	
viking hypomorph			**0.40**±0.10		**0.69**±0.16	
tj>mys RNAi			**0.21**±0.06		**0.40**±0.16	
tj>ßPS			**0.37**±0.17		**0.65**±0.26	

The fold change in fluorescence levels between mutant and control egg chambers is shown. Mean and standard deviation of a minimum of 11 and a maximum of 103 measurements per genotype and stage are indicated. See Supplementary Fig. 4 for examples of the original data. P values <0.05 were considered statistically significant (*:P<0.05, **:P<0.005, ***:P<0.001).

SUPPLEMENTAL TABLE 2 (related to Fig. S2)

Actin bundle alignment with respect to the AP axis (%)

**genotype**	**angle (°)**		**egg chamber stage**					
			**germarium**	**S1**	**S2-S3**	**S4**	**S5-S6**	**S7-S8**
**control**								
	-90	-81	*2.96*	*0*	*0*	*0*	*0*	*0*
	-80	-71	*1.48*	*0.57*	*0*	*0*	*0*	*0*
	-70	-61	*0.00*	*1.14*	*1.61*	*0.56*	*0*	*0*
	-60	-51	*0.00*	*2.84*	*1.08*	*1.12*	*0*	*0*
	-50	-41	*0.00*	*1.70*	*1.08*	*2.25*	*0*	*1.61*
	-40	-31	*2.22*	*3.41*	*3.76*	*2.25*	*0.83*	*1.61*
	-30	-21	*3.70*	*7.39*	*5.38*	*6.74*	*6.67*	*0.54*
	-20	-11	*8.15*	*15.91*	*11.83*	*10.11*	*12.50*	*2.69*
	-10	1	*26.67*	*15.34*	*17.74*	*14.04*	*22.50*	*27.42*
	0	9	*26.67*	*19.32*	*9.68*	*18.54*	*25.00*	*39.25*
	10	19	*10.37*	*12.50*	*13.98*	*14.61*	*16.67*	*21.51*
	20	29	*5.19*	*5.68*	*15.59*	*10.11*	*5.83*	*4.30*
	30	39	*1.48*	*6.82*	*11.83*	*6.18*	*4.17*	*1.08*
	40	49	*0.74*	*2.84*	*4.84*	*5.62*	*0.83*	*1.61*
	50	59	*1.48*	*2.27*	*1.08*	*3.37*	*0.83*	*1.61*
	60	69	*8.89*	*1.70*	*0.54*	*2.25*	*2.50*	*0*
	70	79	*0.74*	*0.57*	*0*	*1.12*	*0.83*	*0*
	80	90	*0*	*0*	*0*	*0.56*	*0.83*	*0*
***laminin* hypomorph**								
	-90	-81	*0*	*1.19*	*0.58*	*0*	*0*	*0*
	-80	-71	*0*	*1.19*	*0.58*	*0.73*	*0*	*0*
	-70	-61	*0*	*8.33*	*0.58*	*2.92*	*0*	*0.60*
	-60	-51	*0*	*1.19*	*1.16*	*2.92*	*0*	*0.60*
	-50	-41	*1.12*	*1.19*	*1.16*	*2.19*	*0.58*	*0.91*
	-40	-31	*4.49*	*7.14*	*1.16*	*2.19*	*1.16*	*0.91*
	-30	-21	*5.62*	*4.76*	*2.89*	*5.11*	*5.78*	*0.91*
	-20	-11	*8.99*	*8.33*	*6.36*	*5.11*	*9.83*	*3.63*
	-10	1	*19.10*	*17.86*	*11.56*	*10.22*	*23.70*	*5.14*
	0	9	*24.72*	*17.86*	*11.56*	*13.14*	*20.23*	*29.61*
	10	19	*25.84*	*10.71*	*12.14*	*12.41*	*17.92*	*20.85*
	20	29	*7.87*	*10.71*	*15.61*	*8.03*	*6.36*	*23.26*
	30	39	*2.25*	*8.33*	*13.87*	*9.49*	*6.36*	*9.67*
	40	49	*0*	*1.19*	*10.40*	*2.92*	*4.05*	*2.42*
	50	59	*0*	*0*	*4.62*	*8.76*	*1.73*	*1.21*
	60	69	*0*	*0*	*2.31*	*7.30*	*0.58*	*0*
	70	79	*0*	*0*	*2.31*	*2.19*	*1.16*	*0*
	80	90	*0*	*0*	*0*	*2.19*	*0.58*	*0.30*

Quantification of the relative angles of basal actin bundles of follicle cells. A 0° angle represents a bundle perpendicular to the AP axis. Values refer to the percentage of bundles with a given orientation with respect to the AP axis of the ovariole. Intervals that contain at least 9.5% of the scored bundles are highlighted in red. A minimum of 100 bundles were analysed from at least 5 egg chambers per stage.

SUPPLEMENTAL TABLE 3 (related to Fig. S2)

Col IV fibril alignment with respect to the AP axis (%)

genotype	angle (°)		Egg chamber stage	
			S5-S6	S7-S8
**control**				
	-90	-81	*0*	*0*
	-80	-71	*0*	*0*
	-70	-61	*0*	*0*
	-60	-51	*0*	*0*
	-50	-41	*0*	*0*
	-40	-31	*0*	*1.44*
	-30	-21	*1.10*	*1.44*
	-20	-11	*4.40*	*2.01*
	-10	1	*7.69*	*8.91*
	0	9	*32.97*	*34.48*
	10	19	*19.78*	*27.30*
	20	29	*24.18*	*19.25*
	30	39	*5.49*	*4.89*
	40	49	*3.30*	*0.29*
	50	59	*1.10*	*0*
	60	69	*0*	*0*
	70	79	*0*	*0*
	80	90	*0*	*0*
***laminin* hypomorph**				
	-90	-81	*0*	*0*
	-80	-71	*0*	*0*
	-70	-61	*0*	*0*
	-60	-51	*0*	*0*
	-50	-41	*0*	*0.58*
	-40	-31	*0*	*0.58*
	-30	-21	*3.90*	*0.58*
	-20	-11	*2.60*	*2.89*
	-10	1	*6.49*	*5.20*
	0	9	*36.36*	*36.42*
	10	19	*12.99*	*20.81*
	20	29	*12.99*	*24.86*
	30	39	*7.79*	*8.09*
	40	49	*5.19*	*0*
	50	59	*3.90*	
	60	69	*3.90*	*0*
	70	79	*3.90*	*0*
	80	90	*0*	*0*

Quantification of the relative angles of Collagen IV:GFP fibrils. A 0° angle represents a fibril perpendicular to the AP axis. Values refer to the percentage of fibrils with a given orientation with respect to the AP axis of the ovariole. Intervals that contain at least 10% of the scored bundles are highlighted in green. A minimum of 50 fibrils were analysed from at least 5 egg chambers per stage.

SUPPLEMENTAL EXPERIMENTAL PROCEDURES

Experimental genotypes

Figure 1
control: *w*; *ubi-GFP FRT40/CyO*
laminin hypomorph: *w/w; l(2)k05404/LanB1*^{28a}
tj>LanB1 RNAi: UAS-dicer/+; tj-Gal4/+; UAS-LanB1 RNAi/tub-Gal80^{ts}

Suppl. Figure 1
tj>LanB1+LanB2 RNAi: UAS-dicer/+; tj-Gal4/UAS-LanB2 RNAi; UAS-LanB1 RNAi/tub-Gal80^{ts}

Figure 2; Suppl. Movies S1 and S2
control: *w*; *Fas3::GFP/+*; *His2Av::mRFP/+*
laminin hypomorph: *w*; *l(2)k05404 Fas3::GFP/LanB1*^{28a} *His2Av::mRFP*
tj>LanB1 RNAi: UAS-dicer/+; tj-Gal4/+; UAS-LanB1 RNAi/tub-Gal80^{ts}

Suppl. Figures S2 and S3
control: *w*; *l(2)k05404, vkg::GFP/+; +/TM2*
laminin hypomorph: *w*; *l(2)k05404, vkg::GFP/LanB1*^{28a} *His2Av::mRFP*
viking hypomorph: y *w/+*; *vkg*^{01209}/ *vkg*^{K07138}; *ry[506]/+*

Figure 3
control: *w*; *ubi-GFP FRT40/CyO*
laminin hypomorph: *w/w; l(2)k05404/LanB1*^{28a}

Figure 4; Suppl. Figure 4; Suppl. Movie S2
control: *w*; *ubi-GFP FRT40/CyO*
control: *w*; *Fas3::GFP/+*; *His2Av::mRFP/+*
laminin hypomorph: *w/w; l(2)k05404/LanB1*^{28a}
laminin hypomorph: *w*; *(l)2k05404 Fas3::GFP/LanB1*^{28a} *His2Av::mRFP*
tj>LanB1 RNAi: UAS-dicer/+; tj-Gal4/+; UAS-LanB1 RNAi/tub-Gal80^{ts}
tj>mys RNAi: w; UAS-mys RNAi/tj-Gal4; tub-Gal80^{ts}*/+*
tj>ßPS: UAS-ßPS/ traffic jam-Gal4; tub-Gal80^{ts}*/+*
tj>LanB1 RNAi+ßPS (control): UAS-dicer/+; tj-Gal4/UAS-GFP; UAS-LanB1 RNAi/tub-Gal80^{ts}
tj>LanB1 RNAi+ßPS: UAS-dicer/+; tj-Gal4/UAS-ßPS; UAS-LanB1 RNAi/tub-Gal80^{ts}

Figure 5
control: *w*; *ubi-GFP FRT40/CyO*
laminin hypomorph: *w/w; l(2)k05404/LanB1*^{28a}
tj>LanB1 RNAi: UAS-dicer/+; tj-Gal4/+; UAS-LanB1 RNAi/tub-Gal80^{ts}

Figure 6; Suppl. Movies S4-6
control: *w*; *Fas3::GFP/+*; *His2Av::mRFP/+*
laminin hypomorph: *w*; *l(2)k05404 Fas3::GFP/LanB1*^{28a} *His2Av::mRFP*
tj>lanB1 RNAi+GFP (control): UAS-dicer/+; tj-Gal4/tub-Gal80^{ts}*; UAS-LanB1 RNAi/UAS-GFP*
tj>lanB1 RNAi+Abi RNAi: UAS-dicer/+; tj-Gal4/tub-Gal80^{ts}*; UAS-LanB1 RNAi/UAS-Abi RNAi*

Figure 7; Suppl. Movie S7
control: *w*; *Fas3::GFP/+*; *His2Av::mRFP/+*
laminin hypomorph: *w*; *l(2)k05404 Fas3::GFP/LanB1*^{28a} *His2Av::mRFP*
spindle mutant egg chamber from a homozygous female: *w;; ru spn-C*^{094} *e spn-A*^{057} *ca*

Immunohistochemistry

Primary antibodies were used at the following concentrations: rabbit anti-Laminin b1 (Kumagai et al., 1997), 1:1000; FITC-conjugated anti-GFP (Abcam, ab6662), 1:500; anti-Fasciclin3 mouse monoclonal, 1:50 (7G10 anti-Fasciclin III was deposited to the DSHB by Goodman, C.); anti-orb mouse monoclonal, 1:10 (a mixture of Orb 4H8 and Orb 6H4 monoclonal antibodies, 1:20 each; 4H8 and 6H4 anti-Orb were deposited to the DSHB by

Schedl, P.); anti-bPS mouse monoclonal, 1:100 (CF.6G11 anti-βPS was deposited to the DSHB by Brower, D.).

Imaging of ovarian tissues

3-D images of fixed samples were acquired using a 40x/1.3 NA oil immersion objective. 4-D *in vivo* images were acquired at room temperature (~20°C). We used a Leica SP5 confocal microscope controlled by the Leica LAS AF software. To analyse rotation, we performed live imaging of complete ovarioles using a 20x/0.7 NA oil immersion objective and Leica hybrid detectors (standard mode), with time points every 2-4 minutes for 1-6 hours. Follicle cell migration was analysed either with the Fas3::GFP and His2Av::mRFP fluorescence proteins or with transmitted light (phase contrast). Rendering of 4-D live images were performed in Imaris. Snapshots were obtained and processed using ImageJ and Adobe Photoshop, and labelled in Adobe Illustrator.

Transmission Electron Microscopy (TEM)

Ovaries were dissected in Phosphate Buffer Saline + 0.1% Tween-20 and fixed for 2 hours at 4 ^{0}C in 3% glutaraldehyde/l% paraformaldehyde (vol./vol.) in 0.05 M cacodylate buffer (pH 7.4). After three 10 min. washes in cacodylate buffer 0.1 M at 4 ^{0}C, ovaries were postfixed for 1 hour at 4ºC in darkness in 1% OsO_4, 1% $K_3Fe[CN]_6$ in water. After three rinses in distilled water at 4ºC, ovaries were stained for 2 hours at room temperature (RT) in darkness in 0.5% uranyl acetate, rinsed again in distilled water and dehydrated through an ethanol rising series (50%, 70%, 90% and 3x100%; 10 min. each) at RT and infiltrated with Embed 812 resin (Electron Microscopy Sciences). The resin-embedded specimens were polymerized by incubation in fresh Embed 812 during 48 hours at 60 ^{0}C in flat plastic embedding molds, which were cut in 50-70 nm thick sections with a DIATOME diamond-blade fixed on a Reichert Jung Ultramicrotome and mounted on copper grids. Sections were counterstained with 1% uranyl acetate in 50% ethanol for 1 min. and then stained with lead citrate for 5 minutes in a CO_2-free atmosphere. Sections were examined with a Zeiss EM902 electron microscope at 80Kv, and photographed at 50.000x magnification.

Atomic Force Microscopy measurements

Ovarioles were dissected out of the muscle sheath to make sure that the AFM cantilever was in direct contact with the basement membrane. Stiffness of *ex-vivo* ovarian tissues immobilized on Petri dishes using Cell-Tak (BD Biosciences, Oxford, UK) and containing *Drosophila* culture media was tested by AFM within 30 minutes of dissection. Monodisperse polystyrene beads (diameter 5.46 ± 0.12 μm, microParticles GmbH, Berlin, Germany) were glued to silicon cantilevers with a nominal spring constant of 0.1 N/m (PPP-BSI, Nanosensors, Neuchatel, Switzerland). Cantilevers were mounted on a JPK Nanowizard Cellhesion 200 (JPK Instruments AG, Berlin, Germany) set up on an inverted optical microscope. Exact cantilever spring constants were determined using the thermal noise method included in the AFM software. Freshly dissected samples were measured under visual control (Franze et al., 2011). Force-distance-curves were taken at an approach speed of 10 μm/s and a maximum force F = 6 nN. Force–distance curves were analyzed for an indentation depth d = 0.2 μm using a custom algorithm based in Matlab (Christ et al., 2010) (MathWorks, Natick, USA), which fits the Hertz model to the data: $F = \frac{4}{3} K \sqrt{R} \delta^{3/2}$, where the apparent elastic modulus $K = \frac{E}{1-\nu^2}$ is a measure of elastic stiffness, R is the radius of the indenter, E is the Young's modulus, and n is the Poisson's ratio. Normal distribution of AFM measurements was confirmed using the Kolmogorov-Smirnov test. The statistical significance of the differences between experimental and control values was evaluated using two-tailed t-tests. 58 measurements from 11 control ovarioles and 48 from 10 *laminin* mutants were collected.

Quantification of migration speed and fluorescent levels

The tracking of individual cells was performed using Fasciclin3::GFP (Fas3::GFP), Histone2Av::RFP (His2Av:mRFP) or transmitted light (Movies S1-S11). Lineal velocity of follicle cells was calculated by manually tracking nuclei or geometrical cell centres using the Leica LAS AF software, which allowed precise determination of time and length of movement. Dispersion of the migration speed data was represented using box-plot graphs, where the median and the outliers are indicated.

To quantify fluorescence in control and experimental samples and to avoid unwanted variability, both genotypes were dissected in parallel and fixed and immunostained in the same tube. To distinguish between experimental and control samples in a given experiment, we expressed a fluorescent marker (either nuclear GFP or His2Av::mRFP) in only one of the genotypes. In addition, quantification was performed on images captured using identical confocal settings. Z-sections were taken at 0.8-1.0 μm intervals. Colour depth was set to 12-bit and configured so that most of the pixels were within the dynamic range of the detectors. Z-Depth was selected manually and FITC, GFP or Cyanine5 fluorescence intensity signals were quantified in the plane of maximum

signal of the BM (laminin and Col IV) or of the basal surface of the follicle cells (integrin). Analysis was only done on consecutive egg chambers of ovarioles outside of the muscle sheath and from at least three biological replicas. Fluorescent intensity was measured in five different areas per 3+ egg chambers (Fig. S5). Image stacks were converted to grey scale and pre-processed using the standard background subtraction function of ImageJ (default settings; 50 pixel radius). Measurements were taken using IMARIS software with the "Measurement points" tool and/or the "ROI measurement" tool from the ImageJ software choosing the Z plane with maximal fluorescence signal. The "ROI measurement" tool allowed us to calculate the mean grey values for each area (Fig S5). Mean grey value equals the sum of the grey values of all the pixels in the selected area divided by the number of pixels and it is expressed in arbitrary units (A.U.). Egg chambers were staged according to (Spradling, 1993). Basically, we used the shape of the chamber, the morphology of the follicular epithelium and stalk cells, chromatin organisation in the nurse cells and the oocyte/nurse cells size ratio to assign the different stages.

Quantification of basal actin bundle and Col IV fibril alignment

One-plane confocal images were acquired and rotated to orientate the AP axis of the ovariole so that anterior is to the left. The orientation of both types of filaments was calculated by determining the angle between each filament and the AP axis of the ovariole (in the germarium and S1 follicles), or the axis defined by the polar cells (from S2-S8), using the line ROI tool of FIJI (Schindelin et al., 2012). Angular data was distributed from -90° to 90°, being 0° a perpendicular alignment. Angles were grouped in 10° intervals, and the percentage of filaments included within each interval was plotted as radial diagrams. Actin bundle alignment was determined from the germarium to S8 follicles; Col IV (*vkg::GFP*) fibrils were quantified in S5-S8 egg chambers.

Supplemental References

Christ, A.F., Franze, K., Gautier, H., Moshayedi, P., Fawcett, J., Franklin, R.J., Karadottir, R.T., and Guck, J. (2010). Mechanical difference between white and gray matter in the rat cerebellum measured by scanning force microscopy. Journal of biomechanics 43, 2986-2992.

Franze, K., Francke, M., Günter, K., Christ, A.F., Körber, N., Reichenbach, A., and Guck, J. (2011). Spatial mapping of the mechanical properties of the living retina using scanning force microscopy. Soft Matter 7, 3147.

Kumagai, C., Kadowaki, T., and Kitagawa, Y. (1997). Disulfide-bonding between Drosophila laminin β and γ chains is essential for α chain to form αβγ trimer. FEBS Lett 412, 211-216.

Schindelin, J., Arganda-Carreras, I., Frise, E., Kaynig, V., Longair, M., Pietzsch, T., Preibisch, S., Rueden, C., Saalfeld, S., Schmid, B., et al. (2012). Fiji: an open-source platform for biological-image analysis. Nat Meth 9, 676-682.

Spradling, A. (1993). Developmental genetics of oogenesis. In The Development of Drosophila melanogaster, M. Bate, and A. Martinez-Arias, eds. (New York: Cold Spring Harbor Laboratory press), pp. 1-70.
