# Supplementary figures and images for: Laminin Levels Regulate Tissue Migration and Anterior-Posterior Polarity during Egg Morphogenesis in Drosophila
Source: Cell Rep. 2017 Jul 5;20(1):211–23. doi: 10.1016/j.celrep.2017.06.031 (PMC5507772; doi:10.1016/j.celrep.2017.06.031)

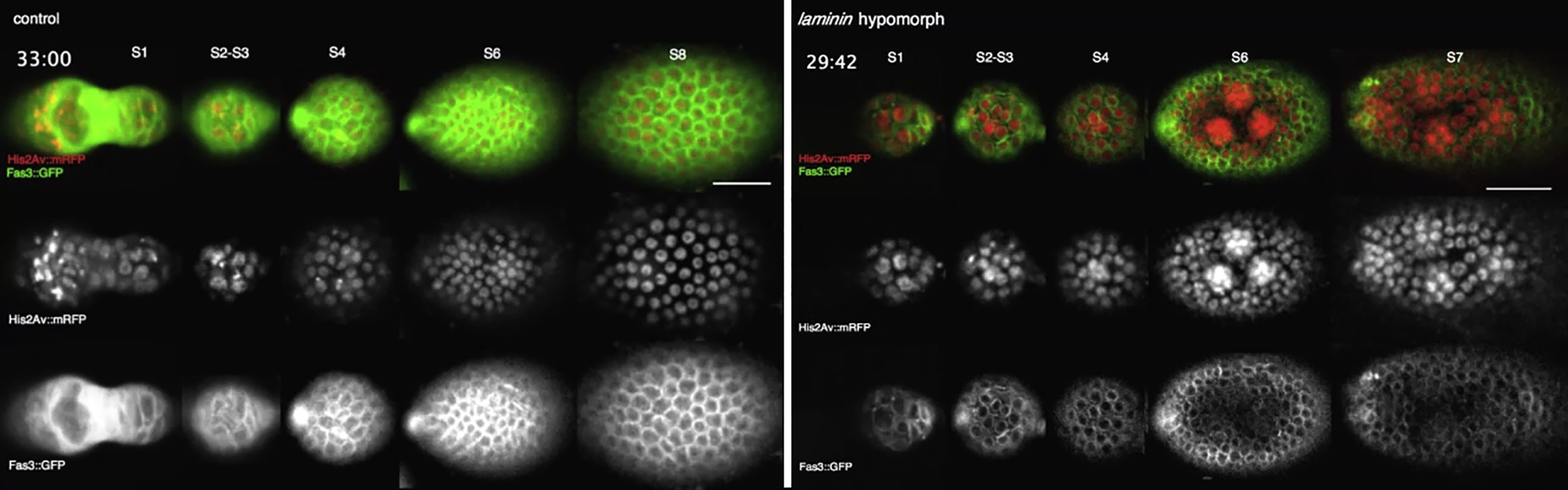

Supplement: Movie S1. Egg Chamber Rotation in Control and laminin Hypomorphic Ovarioles, Related to Figure 2 [file mmc2.jpg]

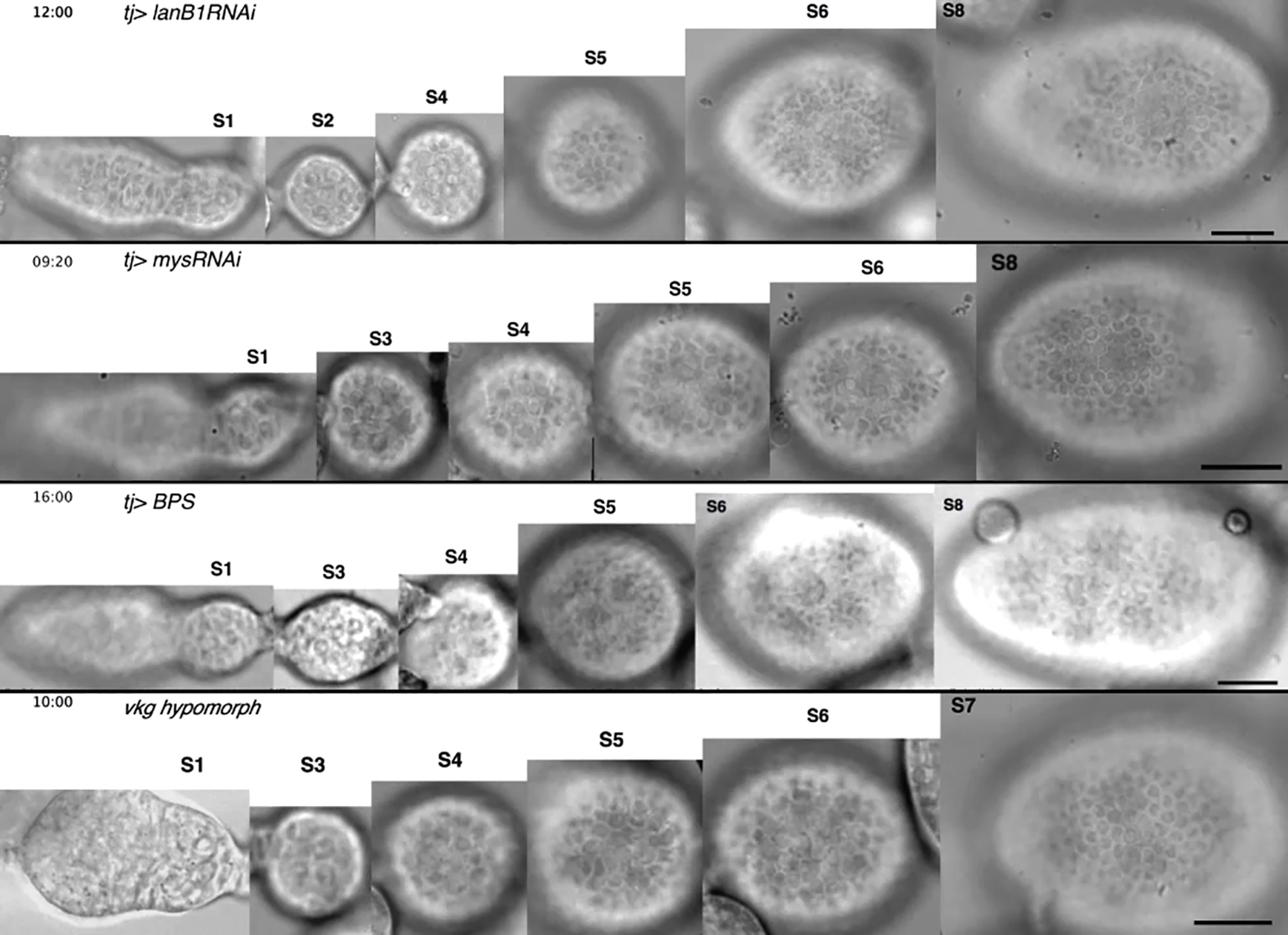

Supplement: Movie S2. Egg Chamber Rotation in tj>LanB1 RNAi, tj>mjs RNAi, tj>βPS and vkg Hypomorphic Ovarioles, Related to Figures 2 and 4 [file mmc3.jpg]

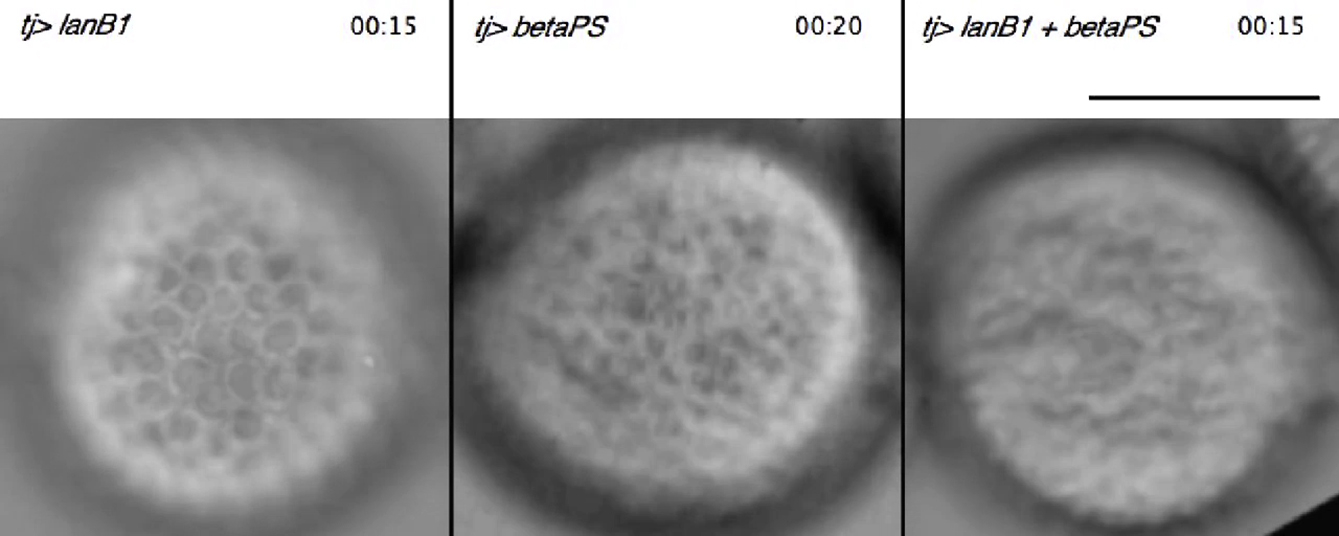

Supplement: Movie S3. Egg Chamber Rotation in tj> LanB1 RNAi, tj>βPS and tj> LanB1 RNAi + βPS Ovarioles, Related to Figure 4 [file mmc4.jpg]

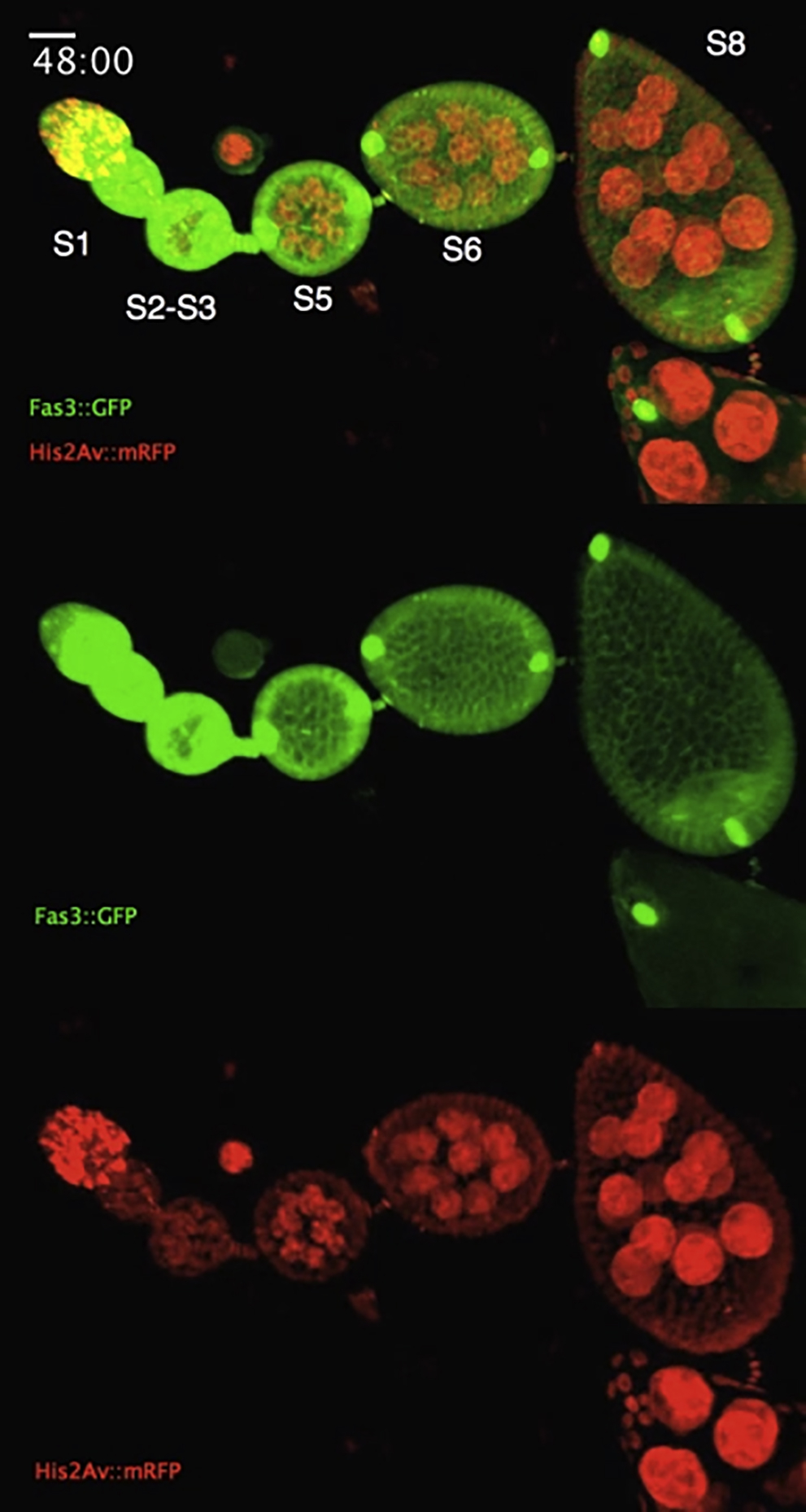

Supplement: Movie S4. Egg Chamber Rotation in a Control Ovariole, Related to Figures 6 and 7 [file mmc5.jpg]

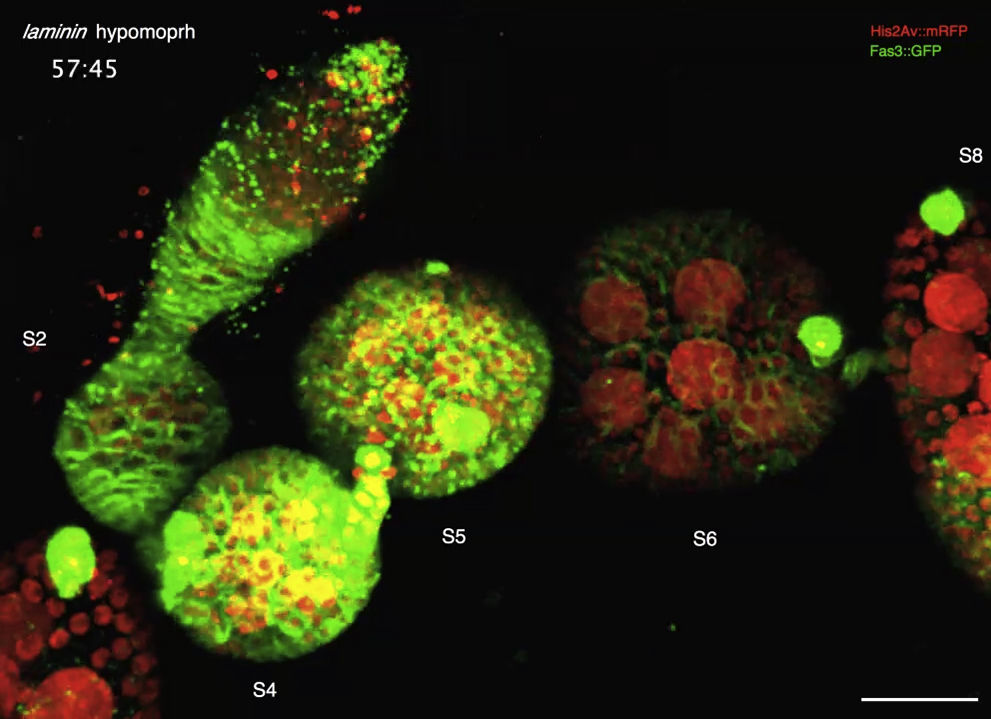

Supplement: Movie S5. Egg Chamber Rotation in a laminin Hypomorphic Ovariole Carrying a Misaligned Egg Chamber, Related to Figure 6 [file mmc6.jpg]

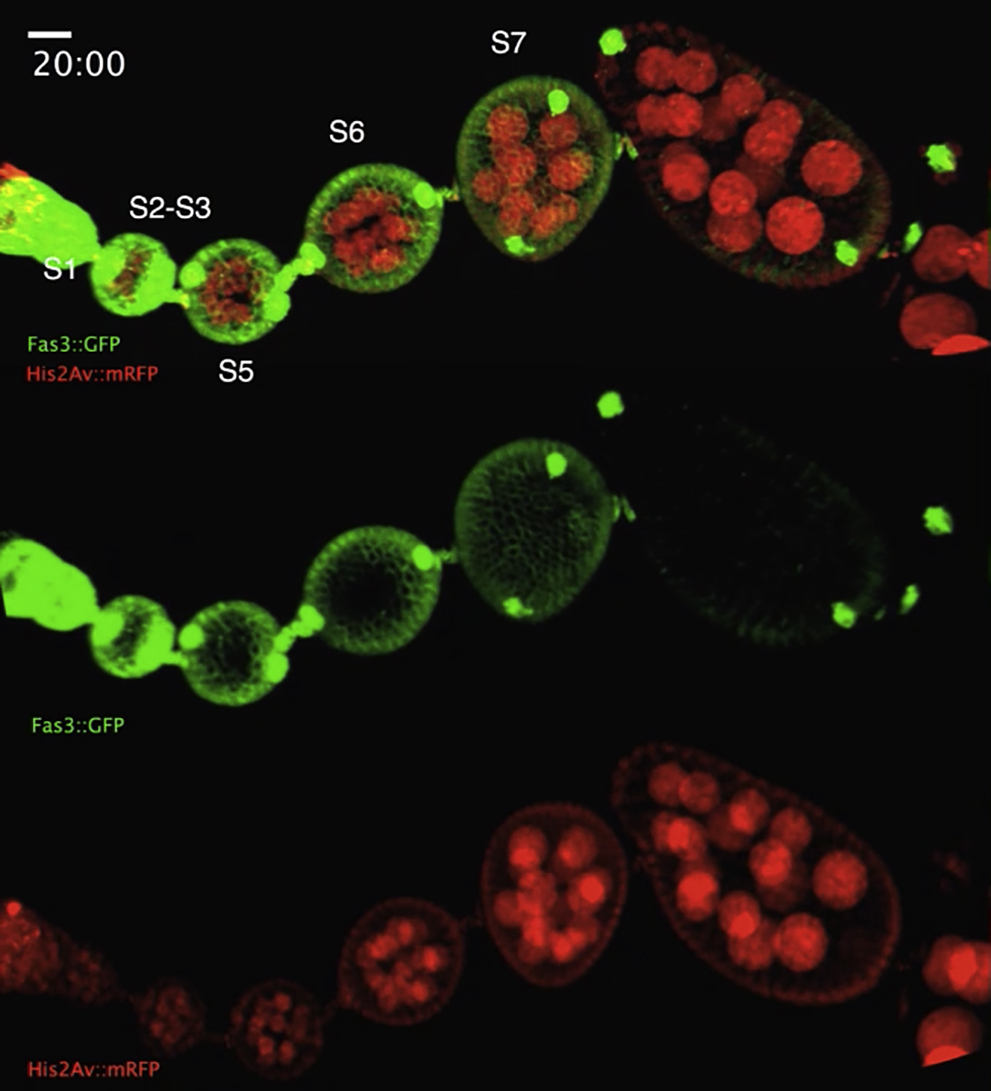

Supplement: Movie S6. Egg Chamber Rotation in a laminin Hypomorphic Ovariole, Related to Figure 7 [file mmc7.jpg]

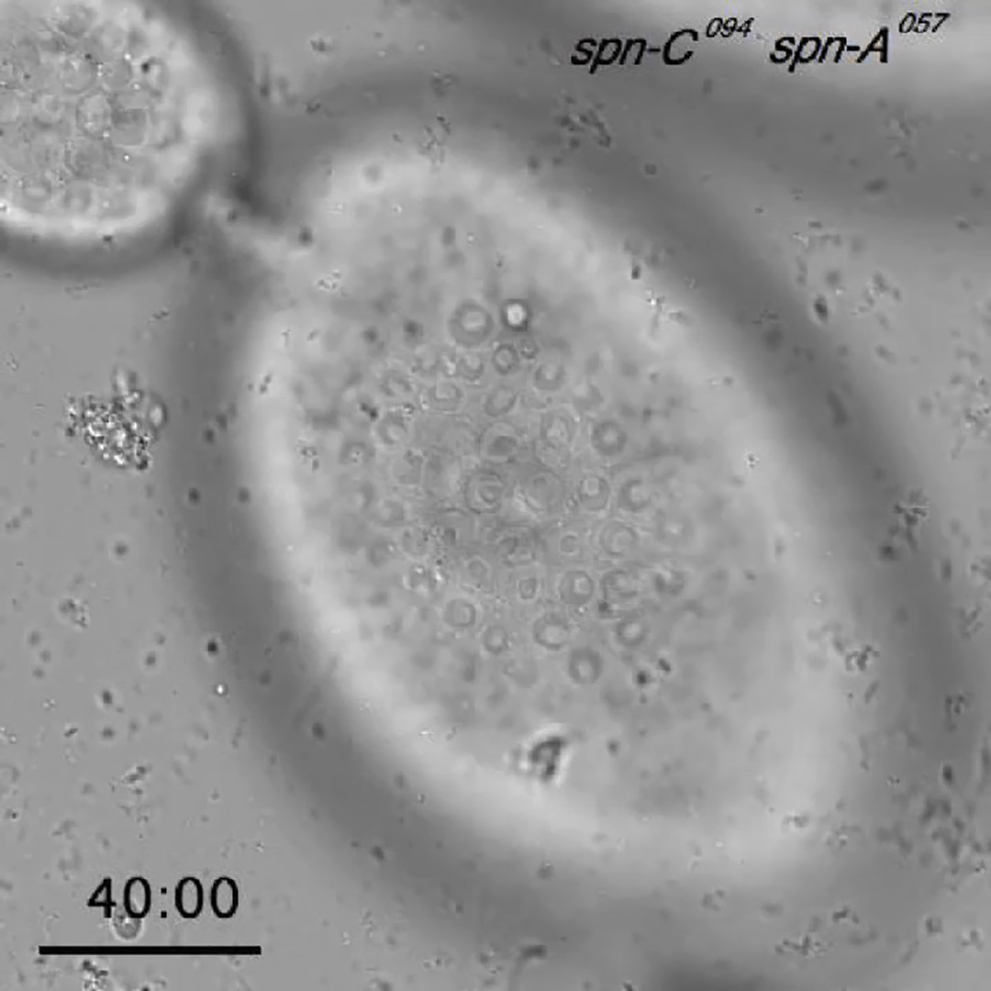

Supplement: Movie S7. Rotation of a spn-C spn-A Double Mutant Egg Chamber Carrying a Misplaced Oocyte, Related to Figure 7 [file mmc8.jpg]
