# Supplementary material for: Laminin Levels Regulate Tissue Migration and Anterior-Posterior Polarity during Egg Morphogenesis in Drosophila
Source: Cell Rep. 2017 Jul 5;20(1):211–23. doi: 10.1016/j.celrep.2017.06.031 (PMC5507772; doi:10.1016/j.celrep.2017.06.031)
Supplement: Document S2. Article plus Supplemental Information [file mmc9.pdf]

Cell Reports

Article

Laminin Levels Regulate Tissue Migration and Anterior-Posterior Polarity during Egg Morphogenesis in *Drosophila*

Graphical Abstract

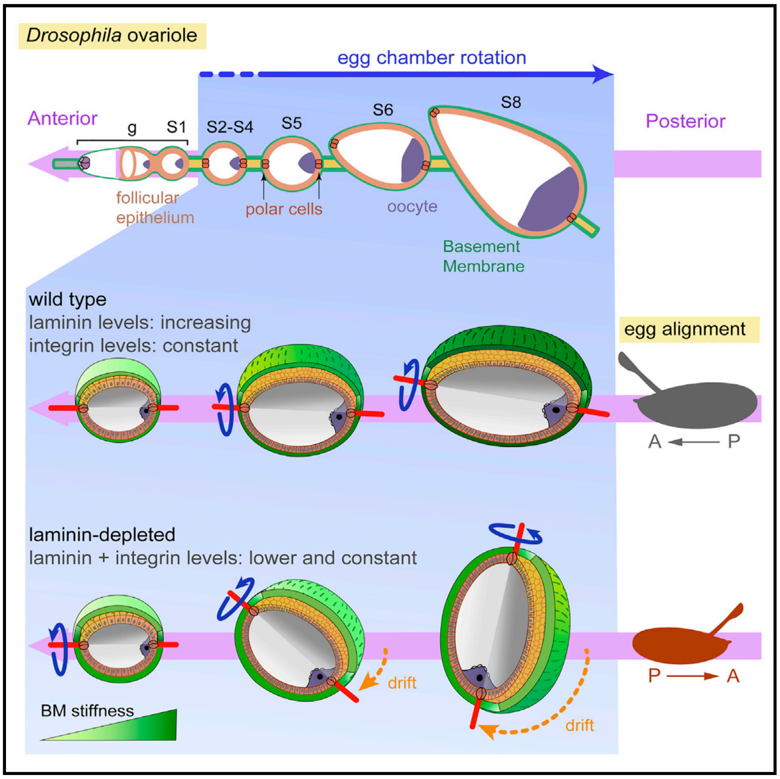

Highlights

- Follicle cells with constant integrin levels face increasing laminin in fly oogenesis
- Integrin-laminin levels fix the timing and speed of egg chamber rotation in vivo
- Laminin depletion affects the ultrastructure and biophysical properties of the ECM
- Laminin depletion results in anterior-posterior misorientation of developing follicles

Authors

María C. Díaz de la Loza,
Alfonsa Díaz-Torres, Federico Zurita, ...,
Kristian Franze,
María D. Martín-Bermudo,
Acaimo González-Reyes

Correspondence

mdmarber@upo.es (M.D.M.-B.),
agonrey@upo.es (A.G.-R.)

In Brief

Collective cell migration requires cell-ECM interactions. Using the fruit fly ovary, Díaz de la Loza et al. find that the ECM component laminin controls the onset and speed of epithelial sheet migration. Because laminin depletion also results in aberrant anterior-posterior polarity, laminin regulates coordinated migration during organogenesis and maintains axial polarity.

CrossMark

Díaz de la Loza et al., 2017, Cell Reports *20*, 211–223

July 5, 2017 © 2017 The Authors.

http://dx.doi.org/10.1016/j.celrep.2017.06.031

Cell Reports
Article

OPEN ACCESS
CellPress

Laminin Levels Regulate Tissue Migration and Anterior-Posterior Polarity during Egg Morphogenesis in *Drosophila*

María C. Díaz de la Loza,[1,4] Alfonsa Díaz-Torres,[1] Federico Zurita,[2] Alicia E. Rosales-Nieves,[1] Emad Moeendarbary,[3,5] Kristian Franze,[3] María D. Martín-Bermudo,[1,*] and Acaimo González-Reyes[1,6,*]
[1]Centro Andaluz de Biología del Desarrollo, CSIC/Universidad Pablo de Olavide/JA, Carretera de Utrera km 1, 41013 Sevilla, Spain
[2]Departamento de Genética e Instituto de Biotecnología, Universidad de Granada, Centro de Investigación Biomédica, 18071 Granada, Spain
[3]Department of Physiology, Development and Neuroscience, University of Cambridge, Downing Street, Cambridge CB2 3DY, UK
[4]Present address: Epithelial Biology Laboratory, The Francis Crick Institute, 1 Midland Road, London NW1 1AT, UK
[5]Present address: Department of Mechanical Engineering, University College London, London WC1E 7JE, UK
[6]Lead Contact
*Correspondence: mdmarber@upo.es (M.D.M.-B.), agonrey@upo.es (A.G.-R.)
http://dx.doi.org/10.1016/j.celrep.2017.06.031

SUMMARY

Basement membranes (BMs) are specialized extracellular matrices required for tissue organization and organ formation. We study the role of laminin and its integrin receptor in the regulation of tissue migration during *Drosophila* oogenesis. Egg production in *Drosophila* involves the collective migration of follicle cells (FCs) over the BM to shape the mature egg. We show that laminin content in the BM increases with time, whereas integrin amounts in FCs do not vary significantly. Manipulation of integrin and laminin levels reveals that a dynamic balance of integrin-laminin amounts determines the onset and speed of FC migration. Thus, the interplay of ligand-receptor levels regulates tissue migration in vivo. Laminin depletion also affects the ultrastructure and biophysical properties of the BM and results in anterior-posterior misorientation of developing follicles. Laminin emerges as a key player in the regulation of collective cell migration, tissue stiffness, and the organization of anterior-posterior polarity in *Drosophila*.

INTRODUCTION

Basement membranes (BMs) are specialized types of extracellular matrix (ECM) that coat the basal side of epithelial and endothelial tissues, surround muscles and fat cells, and play an active role in tissue and organ morphogenesis (Morrissey and Sherwood, 2015). Most BMs are composed primarily of the secreted glycoproteins laminin, type IV collagen (Col IV), nidogen/entactin, and the heparan sulfate proteoglycan perlecan. Alternative proteins that can be found in BMs are papilin, BM-40, and glutactin (Hohenester and Yurchenco, 2013; Yurchenco, 2011). During morphogenesis, BM composition is dynamic, and it changes in a temporal and tissue-specific manner to help sculpt organs and tissues (Huang et al., 2003; Rasmussen et al., 2012; Urbano et al., 2009; Haigo and Bilder, 2011; Harunaga et al., 2014; Pastor-Pareja and Xu, 2011). However, the specific functions of the different BM components during morphogenesis remain uncertain.

Egg development in *Drosophila melanogaster* provides an excellent model system to study in vivo BM contribution to organogenesis. Adult ovaries are composed of tube-producing eggs, termed ovarioles, that show a clear anterior-posterior (AP) polarity. At the anterior tip of each ovariole, a structure called the germarium sustains the continuous production of new follicles. Follicles (or egg chambers) consist of 16-germline cell cysts (15 nurse cells and one oocyte) surrounded by the monolayer follicular epithelium. The oocyte is placed posterior to the nurse cells, a step necessary to polarize the developing egg chamber and to allow proper egg fertilization in the final stages of oogenesis (González-Reyes et al., 1997). During oogenesis, progressively older follicles (classified in 14 stages) remain connected by stalks of specialized follicle cells (FCs) (Spradling, 1993). Stage (S) 1 follicles are found within the posterior third of the germarium. After follicles bud off the germarium and until S4, they adopt a spherical appearance. From S5 to S8, egg chambers elongate along the AP axis, becoming ellipsoid in shape. The AP axis of follicles is aligned with that of the ovariole and is determined by the position of the polar cells (Figure 1A). The apical side of FCs faces the germline, whereas their basal surface contacts a BM containing laminin, Col IV, perlecan, and nidogen (Gutzeit et al., 1991; Lerner et al., 2013; Lunstrum et al., 1988; Schneider et al., 2006). Follicle elongation is generally linked to FC collective migration, a process termed “global tissue rotation” (Cetera et al., 2014; Gates, 2012; Haigo and Bilder, 2011). During this process, FCs project lamellipodia so that the entire egg chamber rotates in a circular trajectory perpendicular to the AP axis without a discernable leading edge and without affecting the AP alignment of follicles. Rotation is accompanied by the polarized secretion of new ECM material, part of which is eventually deposited in fibrils oriented

This is an open access article under the CC BY license (http://creativecommons.org/licenses/by/4.0/).

OPEN ACCESS
CellPress

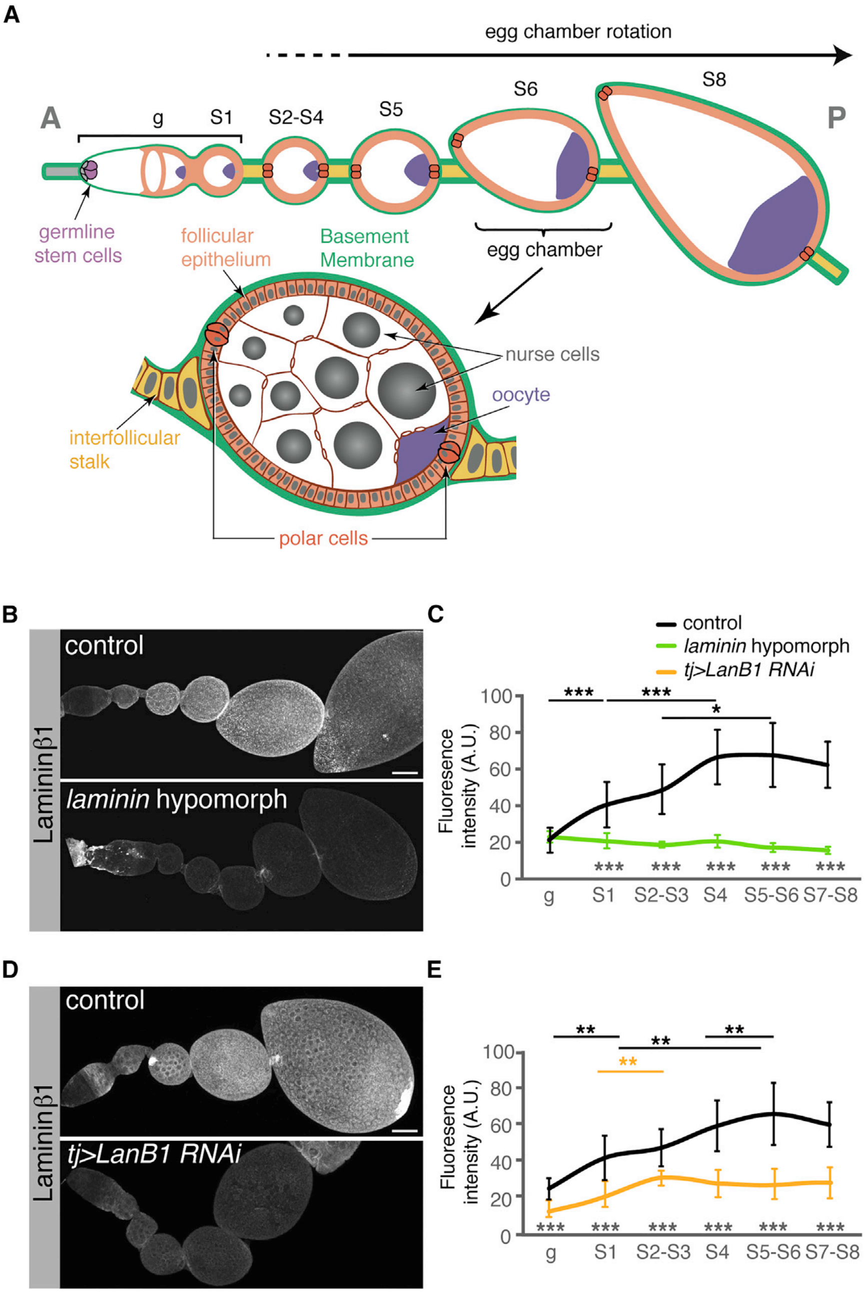

Figure 1. Quantification of Laminin Levels in *laminin*-Depleted Ovaries

(A) Schematic of an ovariole.

(B and D) Immunodetection of the Laminin β1 subunit in control, *laminin* hypomorphic, and *tj > LanB1 RNAi* ovarioles. Mutant conditions show significantly lower Laminin β1 levels. Images are maximum Z projections of at least 15 sections.

(C and E) Quantification of the immunofluorescence signal. Mean and SD of at least 14 measurements per genotype and stage are indicated. Anterior is to the left. g, germarium; S, oogenesis stages. p values of two-tailed t tests < 0.05 were considered statistically significant (*p < 0.05, **p < 0.005, ***p < 0.001). See also Table S1 and Figure S1. Scale bars, 25 μm.

perpendicular to the follicle's AP axis. These fibrils are thought to create a stiffer ECM in the middle region of the follicle that reinforces the planar polarity of the actin bundles, thus allowing more efficient collective migration. As a consequence, the follicle's mid-region offers greater resistance to circumferential expansion than to extension along its polar axis, leading to egg elongation (Bilder and Haigo, 2012; Cetera et al., 2014; Gates, 2012; Horne-Badovinac et al., 2012; Isabella and Horne-Badovinac, 2016). However, it has been recently suggested that egg rotation is not a pre-requisite for elongation because both processes can be uncoupled (Aurich and Dahmann, 2016).

During cell migration, cells adhere and establish traction contacts with their extracellular environment (Friedl and Gilmour, 2009; Reig et al., 2014). Integrins, transmembrane heterodimeric receptors constituted by the non-covalent association of α and β subunits, are the major mediators in cell-ECM interactions (Hynes, 1992). They link the ECM to the actin cytoskeleton to mediate cell movement and to activate different signaling pathways inside the cell (Wehrle-Haller, 2012; Zaidel-Bar and Geiger, 2010). Follicle rotation implicates the interaction of FCs with the ECM via integrins (Haigo and Bilder, 2011). Laminins, main components of the BM, are well-known integrin ligands and consist of single α, β, and γ chains that coil to form a cross shape (Beck et al., 1990; Timpl et al., 1979). When secreted, laminins are proposed to self-assemble through their short arms into networks that recruit the other BM structural components. They remain bonded to the cell surface through the interaction of their long arms with transmembrane receptors, mainly of the integrin family (Hohenester and Yurchenco, 2013; Yurchenco, 2011). Interestingly, the finding that mosaic egg chambers containing laminin-mutant FCs generate round eggs at low frequency suggested that laminins could be involved in FC migration and/or egg chamber elongation (Frydman and Spradling, 2001).

Here we address the role of the balance between integrin and laminin concentrations in epithelial cell migration. By manipulating integrin and laminin levels, we find that the dynamics of laminin-integrin levels regulate the timing and speed of collective migration in vivo. In addition, we unravel a role for BM composition and stiffness in the maintenance of the egg's AP polarity during oogenesis. Because this is a necessary step required

for proper fertilization (Bloch Qazi et al., 2003), our results implicate correct ECM organization in effective fertilization.

RESULTS

Laminin Depletion Results in Premature and Faster Egg Chamber Rotation

Drosophila contains two different laminin trimers composed of either of the two α chains, $\alpha_{3,5}$ (encoded by *LanA*) or $\alpha_{1,2}$ (*wing blister*), and the shared β (*LanB1*) and γ chains (*LanB2*) (Chi and Hui, 1989; Graner et al., 1998; Kusche-Gullberg et al., 1992; Montell and Goodman, 1989). To study the pattern of laminin deposition in the ovary, we utilized an anti-Laminin β1 polyclonal antibody reported to co-localize with both Laminin α subunits in the *Drosophila* embryo and to be specific for Laminin β1 (Kumagai et al., 1997; Urbano et al., 2009). The antibody staining recapitulated Laminin α localization previously described in the germarium and in the BM (Gutzeit et al., 1991; O'Reilly et al., 2008). In summary, Laminin β1 accumulated in the BM that ensheathes the germarium and that is assembled at the basal side of the follicular epithelium from S1 onward (Figures 1B and 1D). Fluorescence quantification revealed that laminin levels doubled from the surface of the germarium to S1 egg chambers when the follicular epithelium is formed and adopts its monolayer appearance. From then on, laminin accumulates progressively in the BM until it reaches maximum values at S5–S6. During S7–S8, the amount of laminin present in the BM decreases slightly with respect to that of S5–S6 egg chambers (Figures 1C and 1E; Table S1).

Making use of the loss-of-function alleles *LanB1*^{28a} and *l(2)k05404* (Urbano et al., 2009), we could obtain a hypomorphic condition for the locus with decreased laminin levels. This viable combination gave rise to adult flies (hereafter referred to as *laminin* hypomorphs) that frequently showed a wing blister phenotype, distinctive of *laminin* $\alpha_{1,2}$ and *laminin* $\alpha_{3,5}$ mutants (Henchcliffe et al., 1993; Martin et al., 1999; Woodruff and Ashburner, 1979). Analysis of *laminin* hypomorphic ovaries confirmed that, from S2–S8, laminin levels were kept constant and significantly lower than those of controls (Figures 1B and 1C; Table S1). A similar reduction in Laminin β1 levels was obtained expressing *LanB1* RNAi in the ovary *(tj > LanB1 RNAi)* (Figures 1D and 1E). These data confirmed that FCs contribute to the laminin deposited in the ovarian BM and that the *laminin* hypomorphic and the *tj > LanB1 RNAi* conditions are useful tools to decrease laminin levels in oogenesis and particularly during follicle rotation.

The fact that maximum values in laminin deposition are observed during follicle elongation at S5–S6, together with the known involvement of integrins in this process, suggested a role for laminin in collective FC migration and egg elongation (Cetera et al., 2014; Haigo and Bilder, 2011). To test this, we severely decreased *LanB1* and *LanB2* expression in the ovary *(tj > LanB1+LanB2 RNAi)* and found that it gave rise to rounder follicles (100%, n = 54) and eggs (86%, n = 89; Figure S1). The appearance of the follicular epithelium was often aberrant, preventing us from assessing in detail the effect of LanB1+LanB2 knockdown on follicle rotation. Thus, laminin is essential for proper egg morphogenesis, a finding supported by the fact that mosaic egg chambers containing FCs mutant for one of the Laminin α chains generated round eggs at low frequency (Frydman and Spradling, 2001). Next, we tracked live FCs to determine whether a milder reduction in laminin function would affect egg chamber rotation and elongation. We used flies carrying *Fasciclin3::GFP* (*Fas3::GFP*) to mark cell membranes and the position of the polar cells and *Histone2Av::RFP* (*His2Av::mRFP)* to label chromatin (Figures 2A and 2B; Movie S1). In agreement with published work (Haigo and Bilder, 2011), all S5–S8 control egg chambers underwent rotation (n = 22). In addition, a significant percentage of S2–S4 egg chambers had also initiated rotation (27.8%, n = 18) (Figure 2C; see also Cetera et al., 2014; Chen et al., 2016). Interestingly, the average rotation speed of control S2–S4 follicles was significantly lower than that of S5–S8 ones (Figure 2D; Table S1; Movie S1), which supports the previous proposition that FC migration is reinforced as rotation proceeds (Cetera et al., 2014; Haigo and Bilder, 2011). The low percentage of motile S2–S4 follicles detected in our movies could be due to the inability of two-thirds of them to sustain migration or to the fact that S2–S4 egg chambers alternated stationary phases with shorter migratory pulses. To distinguish between these possibilities, we imaged live control ovarioles for long periods (3–6 hr). As a positive control for the imaging conditions, we confirmed that all S5–S6 follicles filmed (n = 5) rotated during the entire length of the movies. We filmed S2–S4 follicles (n = 9) for a total of 39.6 hr, 21 of which corresponded to three migrating follicles. Importantly, we never observed rotating follicles that stopped rotation or stationary ones that initiated migration, strongly suggesting that, soon after leaving the germarium, egg chambers either initiate rotation or remain stationary until S5.

laminin hypomorphic and *tj > LanB1 RNAi* egg chambers showed distinctive behavior in terms of rotation initiation and speed. In striking contrast to controls, all of the laminin-depleted S2–S4 follicles underwent rotation (n = 33; Movies S1 and S2). Further, the average rotation speed of mutant follicles was significantly higher than that of S2–S4 controls. Similarly, S5–S8 *laminin* hypomorphs and *tj > LanB1 RNAi* rotated faster than controls (Figure 2D; Table S1). These data confirmed that both the onset and speed of egg chamber rotation in vivo depended on specific laminin levels in the BM. In this regard, the degree of variability in laminin deposition observed in S2–S4 control egg chambers might explain their occasional precocious rotation (Figure 1; Table S1). Finally, in agreement with a recent publication (Chen et al., 2016), none of the S1 egg chambers analyzed from control and laminin-depleted ovaries did rotate (Figures 2A–2C; Movies S1and S2; n = 35).

Laminin Depletion Affects Col IV Deposition and BM Organization and Stiffness

The global alignment of actin bundles at the basal side of FCs into planar-polarized arrays perpendicular to the AP axis is maintained and reinforced during follicle rotation at S5–S8. Concomitantly, newly secreted ECM fibrils build a polarized BM necessary for egg morphogenesis (Cetera et al., 2014; Chen et al., 2016; Gates, 2012; Haigo and Bilder, 2011; Isabella and Horne-Badovinac, 2016; Viktorinová and Dahmann, 2013). We set out to determine whether laminin was required for actin bundle arrangement and for proper BM assembly and deposition.

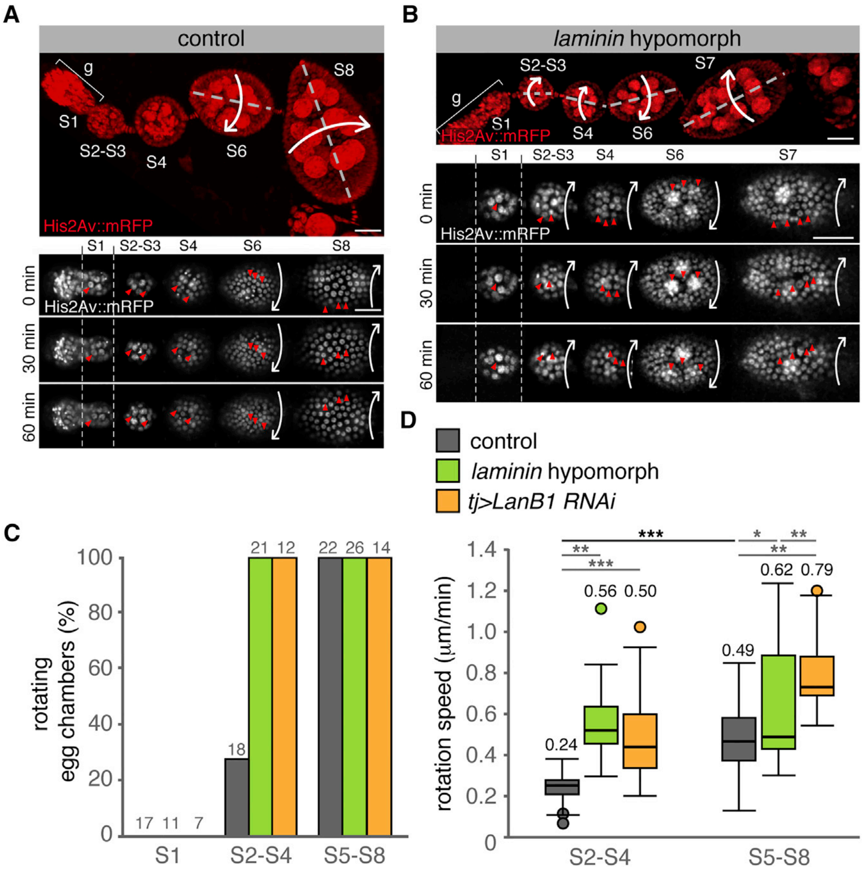

Figure 2. Laminin β1-Depleted Ovarioles Show Premature and Faster Egg Chamber Rotation

(A) Time lapse of a control ovariole.

(B) Time lapse of a *laminin* hypomorphic ovariole.

(C) Quantification of rotating egg chambers in control and Laminin β1-depleted ovarioles. The values above the boxes indicate the number of egg chambers analyzed.

(D) Quantification of the rotation speeds. Horizontal lines in boxes represent median values of a minimum of 23 and a maximum of 103 measurements per genotype and stage. Values above boxes indicate mean rotation speeds.

See also Movie S1 and Table S1 for SD values and Figures S1–S3. Scale bars, 25 µm.

First, we visualized actin filaments with rhodamine-labeled phalloidin to test whether the premature rotation of young *laminin* hypomorphs was caused by an earlier stimulation of actin bundle polarization (Figure S2A; Table S2). Control ovarioles determined that FCs in germarium–S4 follicles (n = 56) display a clear polarization of their basal actin bundles perpendicular to the major axis of the ovariole, even though, in S2–S4 follicles, it is less prominent (Cetera et al., 2014; Chen et al., 2016). Considering that actin bundle alignment is detectable in all analyzed S2–S4 follicles and that only ~30% of them undergo rotation at these stages, the establishment of tissue-level organization of actin bundles is independent of follicle rotation. From S5–S6, actin polarization is gradually enhanced and becomes prominent at S7–S8 (n = 49). The observation of *laminin*-mutant ovarioles yielded similar results, indicating that the reduction in laminin levels typical of the mutant condition did not affect the planar polarity of the basal actin cytoskeleton of FCs (n = 114).

Second, we analyzed whether premature rotation was accompanied by untimely ECM polarization. We made use of a GFP protein trap in the *viking (vkg)* gene, which codes for the α2 chain of Col IV, and looked at Vkg::GFP deposition in control and mutant ovarioles. Although we could detect short Vkg::GFP fibrils in S5–S6 egg chambers, which grew larger and brighter at S7–S8 in both controls and *laminin* hypomorphs, S2–S4 follicles did not present a polarized ECM organization in either genotype (Figure S2B; Table S3; n > 35).

Finally, because the laminin network is essential to recruit other BM components during embryonic development (Urbano et al., 2009), we tested whether laminin acted as a scaffold for the deposition of Col IV in the ovary. As reported before, we found low Col IV levels decorating the germarium, whereas its levels increased gradually until they reach a peak at S7–S8 (Isabella and Horne-Badovinac, 2015). On the contrary, *laminin* hypomorphic follicles accumulated lower levels of Col IV, particularly so in S5–S8 follicles (Figure S3; Table S1). In summary, our results suggest that the main role of laminin in follicle rotation is independent of the establishment of tissue-level planar polarity in migrating FCs. In addition, because precocious migration does not result in fibril orientation prior to S4, continuous follicle rotation is not sufficient for polarized ECM deposition.

To determine in finer detail the consequences for BM organization of laminin depletion, we analyzed BM characteristics using transmission electron microscopy in both control and *laminin* hypomorpic egg chambers. Although control follicles displayed a homogeneous BM ~75–100 nm wide, mutant follicles possessed a wider (~115–135 nm) and less compacted BM (Figures 3A–3C). Next, we utilized atomic force microscopy to measure the apparent elastic modulus K as a proxy for BM stiffness (Figure 3D). To this end, force-distance curves were analyzed for an indentation depth of 200 nm because egg chambers are composite materials with different layers, and structures away from the surface farther than ~1/10 of the indentation depth do not contribute significantly to K (Franze, 2011). We found that control ovarioles are characterized by a significant, continuous increase in BM stiffness soon after follicles leave the germarium. Thus, older follicles possess stiffer BMs than younger ones, at least up to S7–S8. This finding demonstrates a change in BM properties during follicle maturation that coincides with the initiation of consistent tissue rotation. Although laminin-depleted follicles also increased stiffness with time, their K values at S5–S8 were significantly smaller compared with controls. Taken together,

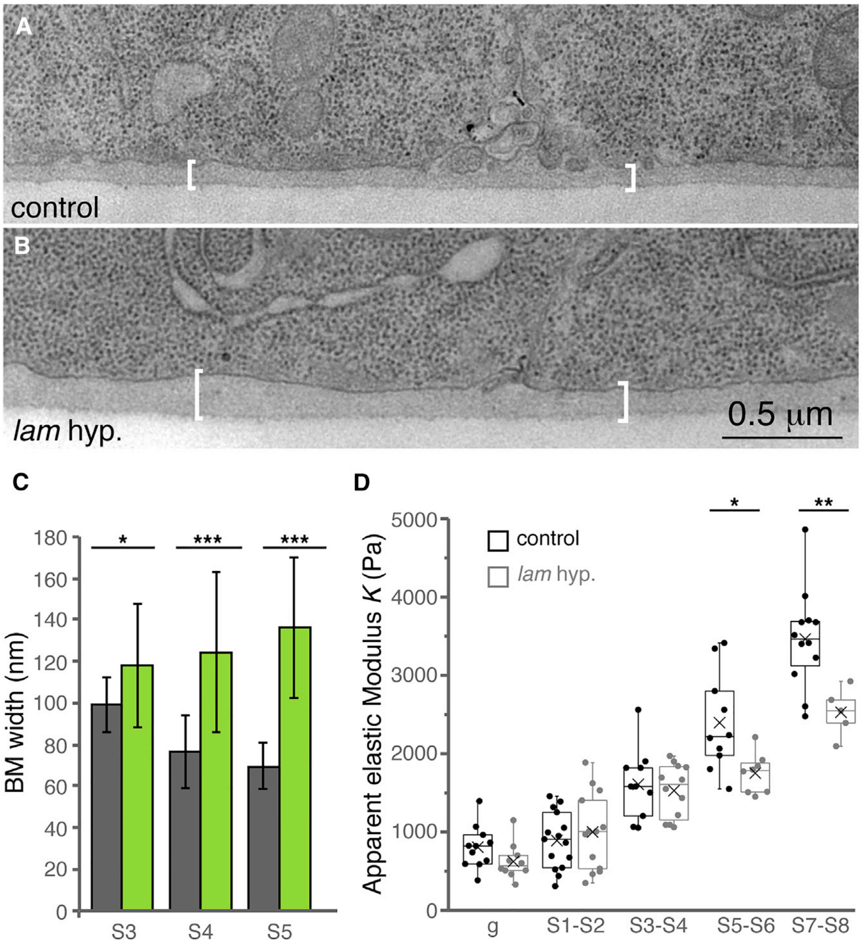

	g/S1-2	S1-2/S3-4	S3-4/S5-6	S5-6/S7-8
control	n. s.	***	**	***
lam hyp.	*	**	n. s.	***

Figure 3. Laminin Depletion Causes Ultra-Structural and Biophysical Changes in the BM

(A and B) Transmission electron microscopy images of the basal side of S4–S5 control (A) and *laminin*-hypomorphic (B) FCs. The BM is indicated with white brackets.

(C) Quantification of BM width. Mean and SD are shown for a minimum of 21 and a maximum of 44 measurements per genotype and stage.

(D) Comparison of the apparent elastic modulus *K* at discrete points along control (58 measurements from 11 ovarioles) and *laminin* hypomorph mutant ovarioles (48 measurements from 10 ovarioles) ex vivo. The results shown refer to an indentation depth of 0.2 μm to reflect BM properties. Statistically significant increases in *K* shown in the graph correspond to control versus mutant tissue of the same stage. The table refers to the comparison between follicles of the same genotype but of different stage. Horizontal lines in boxes represent median values; Xs indicate mean values. n.s., not significant ($p > 0.05$, two-tailed t test).

See also Table S1. Error bars indicate SD.

the lower Col IV levels and the less compacted BM observed in laminin hypomorphs most likely reflect the essential role laminins play in BM assembly because their polymerization is a pre-requisite for BM maturation (Hohenester and Yurchenco, 2013).

Integrin Levels in FCs Influence Laminin Deposition in the BM

Our previous results show that FC migration speed increased concomitant to laminin deposition in control ovarioles. However, the strong laminin reduction characteristic of *laminin* hypomorphic or *tj > LanB1 RNAi* ovarioles results in precocious follicle rotation and increased migration speed. These seemingly opposing results prompted us to analyze the role of integrins in FC migration over the BM. Integrins are the main transmembrane receptors that mediate the cell's response to the BM, connecting the ECM with the actin cytoskeleton and activating different signaling pathways (Wickström et al., 2011; Zaidel-Bar and Geiger, 2010). This interaction is essential during cell migration because integrins are required for the initiation and maturation of adhesion sites, the traction points that allow cell movement through/over the ECM (Galbraith et al., 2007; Wehrle-Haller, 2012). In vitro, cell-substratum adhesion can have opposite effects because it can promote or slow down migration depending on the amount of substrate, integrin expression, or activity and integrin-ligand affinity (Palecek et al., 1997). Indeed, depletion of the integrin β subunit (βPS) in FC clones impairs rotation (Haigo and Bilder, 2011), whereas a milder decrease in integrin levels in *mys*$^{+/-}$ follicles has the opposite effect, increasing rotation speed (Lewellyn et al., 2013). Thus, we investigated whether the changes observed in follicle rotation in response to laminin levels depended on integrin expression in the FCs. To this end, we made use of an anti-βPS antibody to determine integrin levels at the basal surface of FCs from germarial stages to S8 in a number of genetic backgrounds (Figure 4A; Figure S4A; Table S1). The βPS subunit heterodimerizes with αPS1 to form the only functional, laminin-binding integrin expressed from S2–S8 (Devenport and Brown, 2004; Fernández-Miñán et al., 2007). Control ovarioles showed the lowest integrin levels in the anterior half of the germarium, maximum levels in S1 follicles, and then a relatively constant amount from S2–S8. In the *laminin* hypomorph and in *tj > LanB1 RNAi*, integrin levels were also constant from S2–S8, although their levels were consistently lower than those of controls (between 0.4- and 0.8-fold those of controls; Table S1). Thus, laminin depletion reduces the recruitment of integrin receptors to the basal surface of FCs, confirming that the analysis of follicle rotation should take into account the relative levels of laminin and its integrin receptor αPS1βPS.

OPEN
ACCESS
CellPress

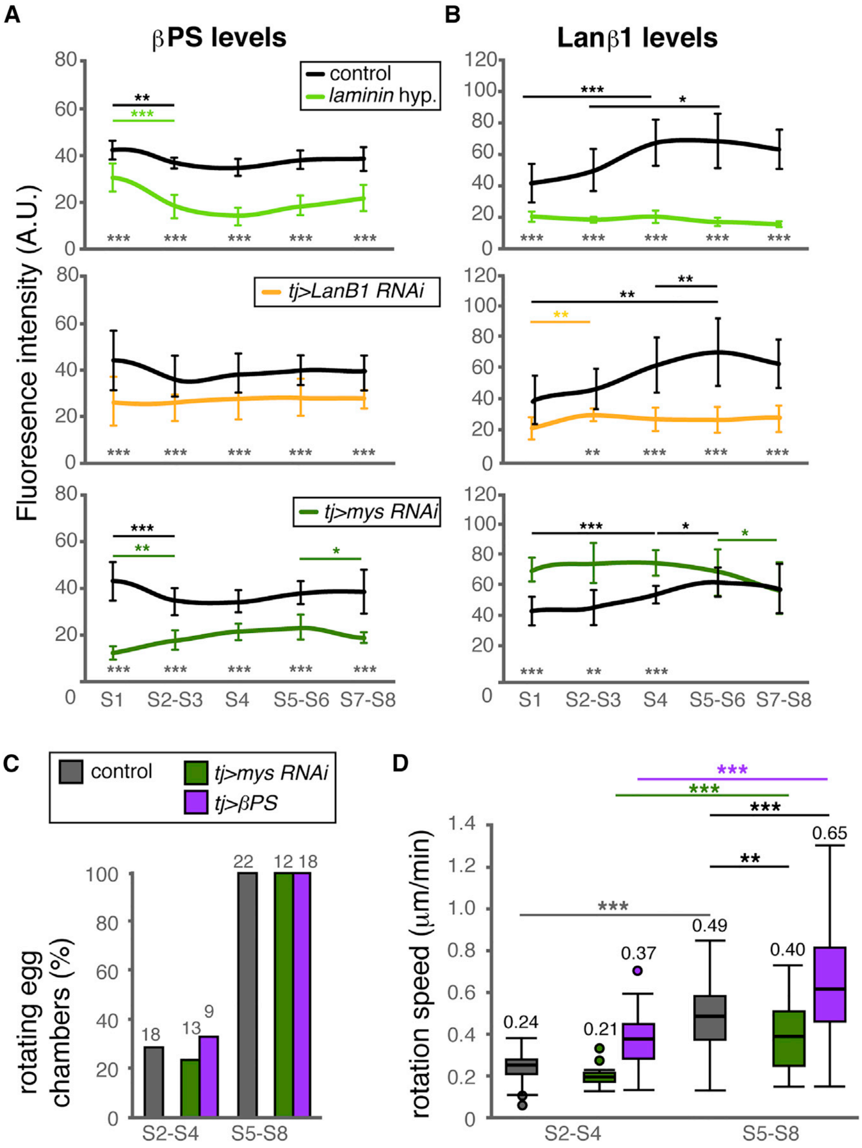

Figure 4. Integrin and Laminin Levels and Rotation Speeds

(A and B) Quantification of the immunofluorescence signal of anti-ßPS (A) and anti-Laminin β1 (B) antibodies. Mean and SD values from three to five ovarioles per genotype are plotted.

(C and D) Onset of rotation (C) and quantification of rotation speed (D) at different stages. Horizontal lines in boxes represent median values of a minimum of 11 and a maximum of 103 measurements per genotype and stage. Values above boxes indicate mean rotation speeds.

See also Figures S4 and S5 and Movies S1 and S2.

We next decreased integrin levels expressing a *mys* RNAi construct (*tj > mys RNAi*) in the follicular epithelium and correlated the new integrin amounts with those of laminin. Quantification of the relative integrin amounts confirmed that, from S2 to S8, *tj > mys RNAi* FCs accumulated lower integrins at their basal side (0.3- to 0.6-fold). Surprisingly, laminin deposition in S1–S4 was consistently increased when basal integrin was reduced in *tj > mys RNAi*, whereas, from S5 to S8, it reached control values (Figure 4; Figure S4; Table S1). These data, together with the decrease in integrin levels observed in the *laminin* hypomorphic and *tj > LanB1 RNAi* conditions, indicated that basal localization of integrins in the follicular epithelium and laminin levels in the adjacent BM are interdependent. Unbeknownst to us, the reason for the higher accumulation of laminin in S1–S4 *tj > mys RNAi* egg chambers, the experimental manipulation of integrin levels in the follicular epithelium, nonetheless provides the means to elucidate the output of laminin-integrin interactions during collective cell migration in an in vivo context.

Laminin Levels in the BM and Integrin Amounts in FCs Dictate the Onset of Rotation and Migration Speed

Next we analyzed the migratory behavior of FCs upon genetic manipulation of laminin and integrin levels and reached two major conclusions. First, the onset of follicle rotation at S5 does not depend only on integrin levels because S2–S3 *tj > mys RNAi* and *laminin* hypomorphic egg chambers contain similar integrin amounts, but the former did not show the precocious migration characteristic of the latter. Further, because laminin levels are very different in the above genotypes, it is likely that the interplay between integrin availability and ECM composition dictates the timing of rotation (Figure 4C; Movie S2; Table S1). Second, cells with similar integrin amounts but facing BMs with different laminin concentrations migrate at markedly different maximum speeds. Thus, S5–S8 *laminin* hypomorphic and *tj > mys RNAi* egg chambers contain similar integrin levels (~0.5- to 0.6-fold those of controls in both genotypes) but different laminin amounts (~17 a.u. *laminin* hypomorh, ~57 a.u. *tj > mys RNAi*). Importantly, they differ in their maximum migration speeds (0.62 μm/min and 0.41 μm/min, respectively) (Figures 2D and 4; Table S1). Altogether, our results strongly suggest that integrin-ligand levels direct the timing and speed of in vivo collective migration. Furthermore, increasing integrin amounts enhanced egg chamber migration speed without affecting the onset of rotation (*tj > ßPS*; Figure 4; Movies S2 and S3), but the combination of high integrin levels with laminin depletion blocked rotation in 86% of the S2–S6 follicles analyzed (n = 7) (*tj > lanB1 RNAi + ßPS*. All *tj > lanB1 RNAi + GFP* S2–S6 controls rotated as expected; n = 12; Movie S3). To emphasize further that finely tuned cell-ECM interactions regulate collective migration in vivo, we found that a reduction in the levels of

the structural BM component Col IV caused precocious and faster follicle rotation (Figures 4C and 4D; Figure S3; Movie S2; Table S1).

Laminin Is Required for Egg Shape and to Maintain AP Axis Alignment

Given their precocious rotation and faster migration speeds, we wished to determine whether egg morphogenesis was affected in *laminin* hypomorphic egg chambers. To this end, we measured the length/width ratio of control eggs, which, in agreement with published data, we found to be ~2.75 (Markow et al., 2009; but see Andersen and Horne-Badovinac, 2016). Mutant eggs laid by *laminin* hypomorphic females did not show any significant differences in length/width ratio compared with control samples (Figures 5A and 5B). Nevertheless, mutant eggs were significantly shorter and thinner than controls, suggesting that proper egg morphogenesis requires a fully functional BM in the ovary.

Careful analysis of laminin-depleted females revealed a phenotype in the organization of the ovariole. The AP axis of developing egg chambers is established early in oogenesis, as shown by the determination of the polar cells at both sides of the developing chamber and by the placement of the oocyte posterior to the sibling nurse cells, in contact with the posterior polar cell cluster (Figure 1A; González-Reyes and St Johnston, 1998). Importantly, because the egg chamber's AP axis is aligned with that of the ovariole throughout oogenesis, mature eggs are also oriented along the AP axis, an arrangement that allows fertilization to occur when the activated egg is lodged into the uterus (Bloch Qazi et al., 2003). In contrast, both *laminin* hypomorphs and *tj > LanB1 RNAi* females displayed a high proportion of egg chambers in which the oocyte was no longer found at the posterior (Figures 5C–5F). These mispositioned oocytes are not due to the reduction in integrin levels characteristic of laminin-depleted follicles because *tj > mys RNAi* females do not contain misaligned follicles (n = 172). Upon closer examination, two aspects of the oocyte mispositioning phenotype were noted. First, oocyte misplacement was evident only from S4 onward, and the phenotype increased with time. Second, follicles with misplaced oocytes seemed to display correct AP polarity because the polar cell clusters were in line with the oocyte. Thus, although the AP axes of follicles were properly established, it was their alignment with the AP axis of the ovariole that was aberrant (Figures 5C and 5D). As a consequence, laminin-depleted ovaries contained eggs with head-to-head or back-to-back orientations as opposed to the canonical back-to-head arrangements found in control ovaries (Figure 5F). Of interest, because *vkg* hypomorphic ovaries showed precocious rotation (see above) but did not contained misplaced oocytes (n = 98), our results demonstrate that laminin, but not Col IV, is required for proper follicle AP axis alignment.

The finding that oocyte mispositioning is detected after germarial stages suggested that rotation of laminin-depleted follicles was necessary for AP axis misalignment. To test this, we studied two different experimental situations. First, we filmed *laminin*-hypomorphic ovarioles, looking for follicles originally aligned with the ovariole's AP axis and that, upon global migration, would deviate their AP axis. We used the position of the posterior interfollicular stalk as a landmark for the original position of the follicle's posterior. In control egg chambers, posterior polar cells and the posterior stalk remained apposed in all cases analyzed. On the contrary, we could detect a shift in the relative position of the posterior polar cells and the adjacent stalk in some laminin-depleted follicles (Figures 6A and 6B; Movies S4 and S5), suggesting that Laminins were essential for proper AP axis alignment by preventing off-axis rotation of developing follicles. Second, we blocked the rotation of laminin-depleted follicles by knocking down the SCAR complex component Abelson-interacting protein Abi (Cetera et al., 2014) and analyzed AP axis orientation. Control *tj > lanB1 RNAi + GFP* follicles showed 11.3% misplaced oocytes (n = 62). Experimental *tj > lanB1 RNAi + Abi RNAi* ovaries displayed a large proportion of malformed follicles in which the follicular epithelium was aberrant. However, in follicles possessing normal-looking epithelia, the oocyte was placed at the posterior (n = 23). Together, the above experiments dictate that a decrease in laminin levels can cause off-axis follicle rotation and, as a consequence, AP axis misalignment.

The Axis of Rotation Is Determined by the Position of the Polar Cells

Live imaging allowed us to detect migrating egg chambers in which their axis of rotation was shifted with respect to that of the ovariole. Because we used the Fas3::GFP line to visualize polar cells in rotating egg chambers, we could determine that the misaligned axes of rotation were in line with the position of the polar cells (Figures 7A and 7B; Movie S6). Therefore, because polar cells organize the planar cell polarity of the follicular epithelium (and, hence, the orientation of lamellipodia) perpendicular to the AP axis (Frydman and Spradling, 2001), polar cells could define the axis of rotation. To test this possibility, we determined that the perpendicular orientation of actin filaments and of Vkg::GFP fibrils was defined by the position of the polar cells in misaligned follicles (Figure 7C). Second, to rule out the possibility that it was the position of the oocyte that fixed the rotation axis, we imaged follicles mutant for *spindle*-group genes in which the polar cells were aligned with the ovariole's AP axis (González-Reyes et al., 1997) but that contained misplaced oocytes. As shown in Figure 7D and Movie S7, these egg chambers rotated according to the axis defined by the polar cells. We conclude that it is the position of polar cells and not that of the oocyte that determines the orientation of the rotation axis, most likely by establishing the planar cell polarity of the follicular epithelium.

DISCUSSION

AP Egg Orientation Depends on Laminin Levels

During normal oogenesis, follicles rotate approximately three complete turns, but their axes of rotation do not deviate from their normal AP alignment. Our results show that maintenance of rotation axis orientation requires proper laminin levels because laminin-depleted ovaries display a significant proportion of misaligned follicles. Although mutant S5–S8 follicles possess BMs with altered elastic properties, it is unclear whether

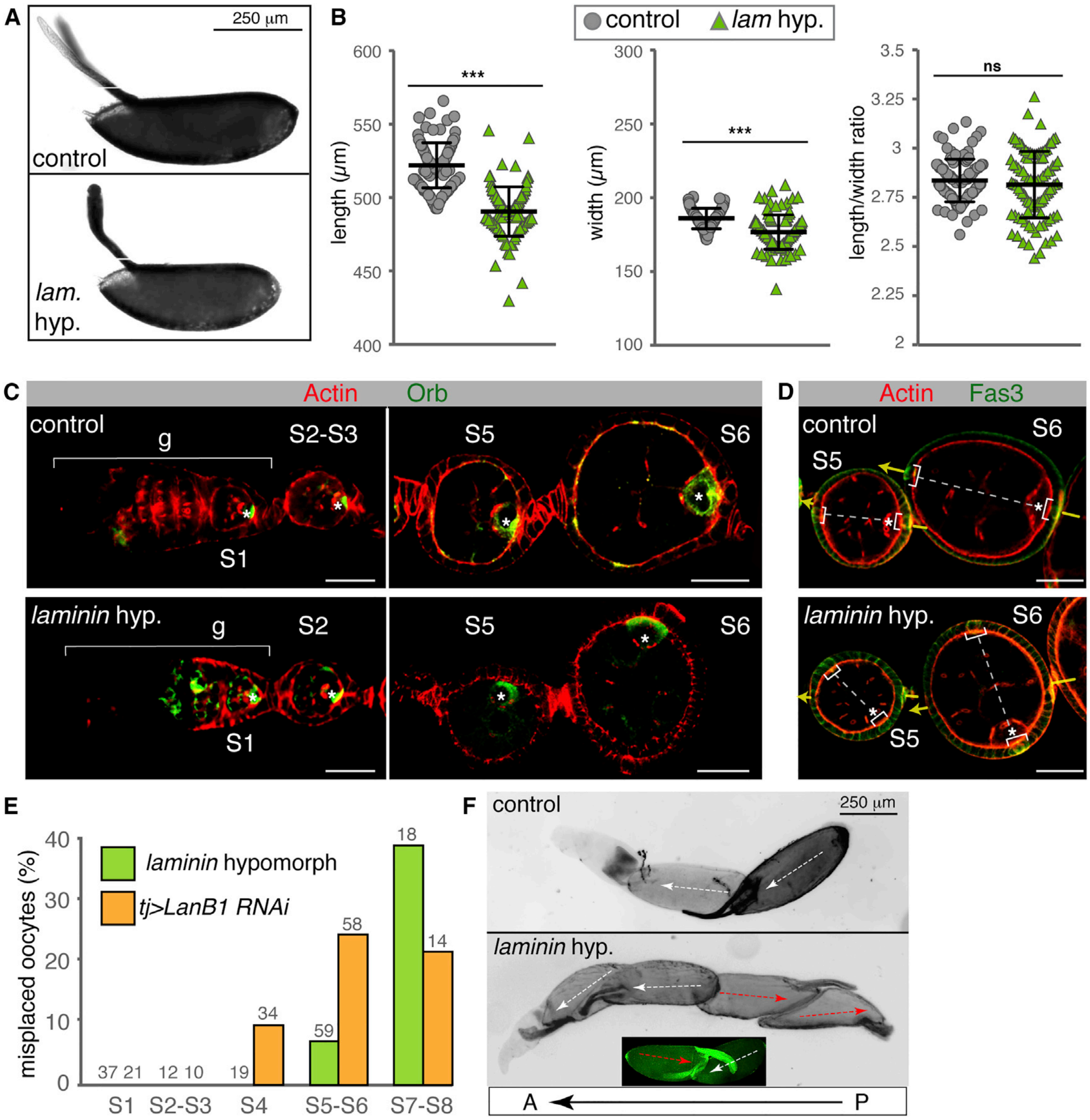

Figure 5. Laminin Depletion Results in Egg Chamber AP Axis Misalignment

(A) Eggs laid by control and *laminin* hypomorphic females.

(B) Quantification of length, width, and aspect ratio. Control eggs: 525 ± 15.5 µm length, 190 ± 6.85 µm width, aspect ratio = 2.74 ± 0.20; *laminin* hypomorphic eggs: 493 ± 16.8 µm length, 181 ± 11.4 µm width, aspect ratio = 2.76 ± 0.13. Mean and SD are shown (n = 100 per genotype). ns, not significant ($p > 0.05$).

(C) The oocyte (labeled in green, white asterisk) is found at the posterior of S1–S4 control and *laminin* hypomorph follicles. At later stages, a fraction of *laminin* hypomorphs shows oocytes misaligned with the ovariole's AP axis.

(D) The Fas3 marker labels the position of the anterior and posterior polar cells (white brackets). In controls, polar cells align with the ovariole's AP axis (yellow arrows). Laminin-depleted egg chambers display polar cells misaligned with the ovariole's axis. In both controls and laminin-depleted follicles, the oocyte is always adjacent to the posterior polar cells.

(E) Quantification of the frequency of misplaced oocytes. Control follicles displayed 100% posterior oocytes (n = 259). The number of follicles analyzed is shown.

(F) Mature eggs found in control ovarioles are always posterior-first (white arrows). *laminin* hypomorphic ovarioles can display mature eggs with a reverse polarity (red arrows), resulting in head-to-head or back-to-back orientations, A, anterior end of ovariole; P, posterior end; asterisks, oocytes.

Scale bars = 25 µm, except in (A) and (F), which are 250 µm.

this causes rotation axis drift. We envisage that the planar-polarized follicle cell-BM interactions show little variability in a properly assembled, stiff ECM, thus fixing the rotation axis and constraining egg chamber movement. The more elastic BM characteristic of laminin-depleted S5–S8 follicles would allow a higher degree of variability in cell-ECM interactions, often resulting in off-axis rotation. This unexpected function of cell-ECM interactions in the maintenance of AP polarity of developing follicles implicates laminins in the final orientation of mature eggs within the ovary. The positioning of the oocyte at the posterior

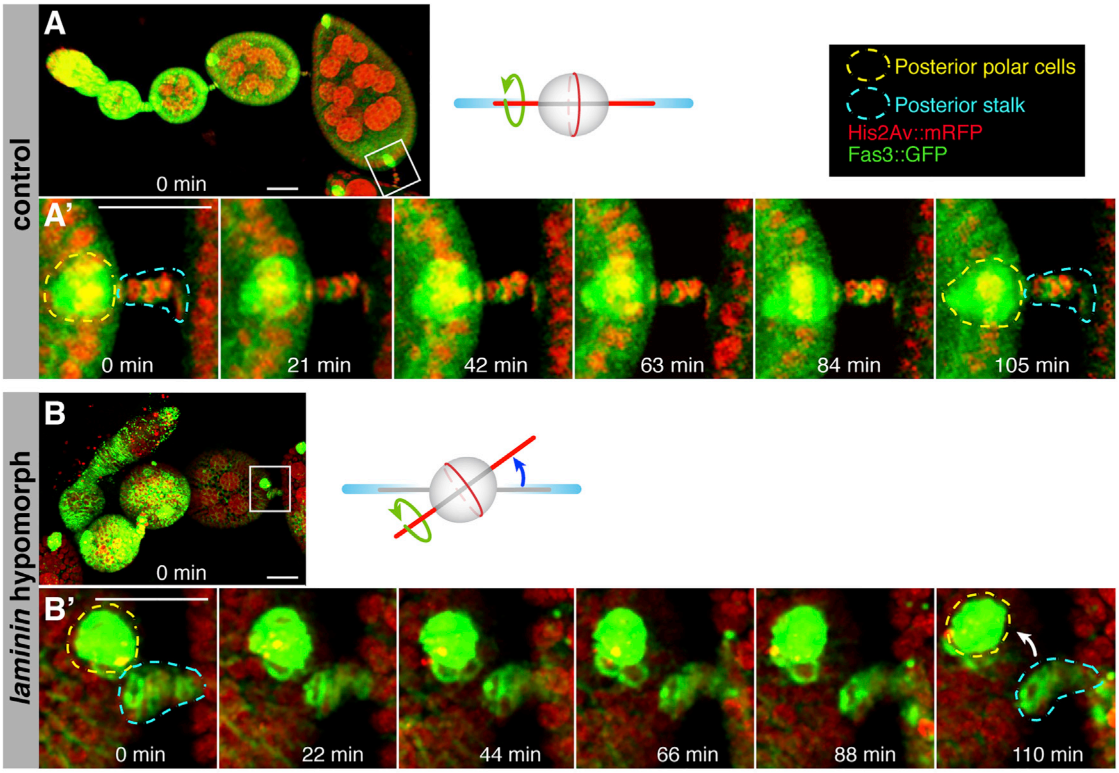

Figure 6. Oocyte Misplacement in Laminin-Depleted Ovaries as a Result of Egg Chamber Rotation

Shown are time lapse movie images of control and *laminin* hypomorphic follicles carrying the histone marker His2Av::mRFP and the polar cell marker Fas::GFP.
(A and A') Controls maintain the alignment of posterior polar cells (yellow dotted line) and the interfollicular stalk (blue dotted line) during rotation.
(B and B') In contrast, in *laminin* hypomorphs, posterior polar cells and stalk cells can separate during rotation. The separation of the posterior polar cells from the stalk is limited (white arrow), but it is worth mentioning that the movie lasts only one-tenth of the total rotation from S5 to S8.
See also Movies S4 and S5. Scale bars, 25 μm.

pole of egg chambers in germarial stages dictates that mature eggs are lodged in the uterus bottom-first. As a consequence, the micropyle (the sperm entry site) faces the sperm storage organs seminal receptacle, spermathecae, and accessory glands, facilitating fertilization (Bloch Qazi et al., 2003). Considering the significant fraction of misaligned follicles found in laminin-depleted ovaries, physiological levels of laminin are likely to influence the fertilization rate in *Drosophila*. Interestingly, the misalignment phenotype characteristic of mutant follicles suggests that the specific laminin function(s) regulating the onset of rotation do(es) not control the rotation axis. The fact that collagen IV-depleted egg chambers also show precocious and faster rotation but do not contain misplaced oocytes supports this idea.

Regulation of Tissue Migration by Cell-ECM Interactions

Mathematical models combined with 2D or 3D cell culture experiments (DiMilla et al., 1991; Huttenlocher et al., 1996; Palecek et al., 1997) suggest that maximum migration speed is reached when integrin occupancy by the extracellular substrate leads to an intermediate adhesive strength in which cell traction—required to trigger cell movement—and cell adhesiveness—which slows down migration—are balanced. Thus, the migration speed of cells containing a normal pool of integrins showed a biphasic response to the amount of substrate, with maximal velocity at middle levels and a block in migration at higher substrate concentrations. In this system, a decrease in integrin levels augmented the amount of substrate required to reach the maximum speed, emphasizing the importance of integrin-ligand levels in the specification of migration parameters in vitro. Our analyses of ligand-receptor interplay during in vivo migration of an epithelial sheet extend these and other observations (Gupton and Waterman-Storer, 2006) and support the following conclusions:

(1) The dynamic interplay in cellular receptor(s)-extracellular ligand(s) levels regulates the *onset* of collective migration. Because both integrins and laminin are essential for rotation, S2–S8 follicles possess constant integrin levels, and their ECM becomes stiffer and contains higher laminin levels with time, we propose that the onset of consistent follicular epithelium migration is governed by changes in the ECM. This property is reminiscent of durotaxis, the ability of cells to follow gradients of matrix stiffness (Lo et al., 2000). Further, because reducing integrin and laminin levels as under the hypomorphic condition initiated

OPEN
ACCESS
CellPress

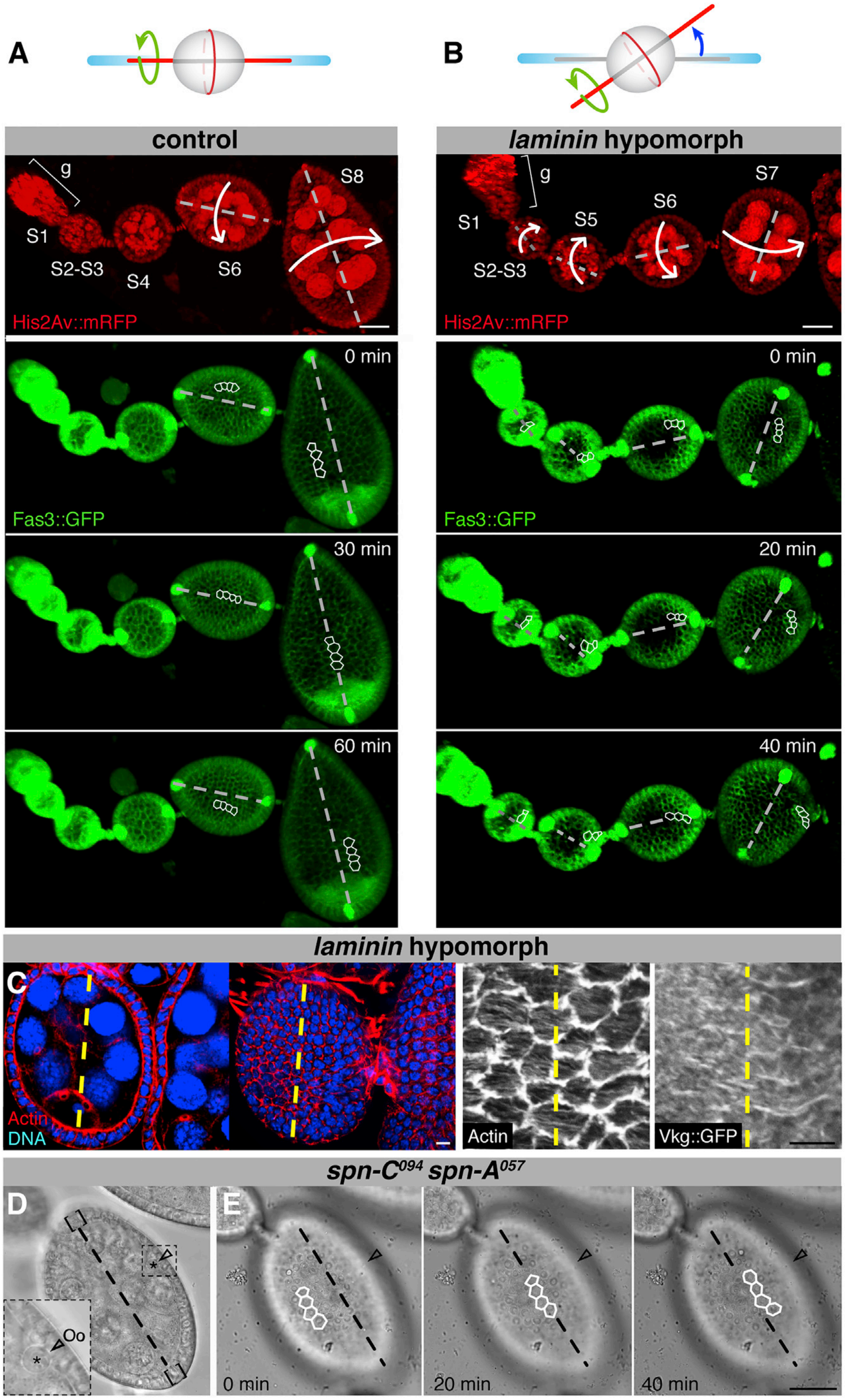

Figure 7. The Position of the Polar Cells, but Not of the Oocyte, Determines the Axis of Rotation

(A and B) Time-lapse analysis of control (A) and *laminin* hypomorphic (B) ovarioles labeled with His2Av::mRFP to mark nuclei and Fas3::GFP to outline FCs and polar cells.

(C) Cross-section and surface view of a misaligned S6 egg chamber from a *laminin* hypomorph carrying the Vkg::GFP protein trap to label ECM fibrils and stained with rhodamine-labeled phalloidin to visualize basal actin filaments.

(D) Phase-contrast image of a *spindle-C spindle-A* double mutant egg chamber carrying a misplaced oocyte, as shown by the position of the oocyte's nucleus (Oo, arrow and asterisk). Brackets mark the presumptive position of the polar cells.

(E) Time-lapse analysis of the rotation axis (dashed line), which is defined by the position of the polar cells.

See also Movies S4, S6, and S7. Scale bars, 25 μm (A, B, and D) and 5 μm. Outlined cells were used to monitor rotation.

rotation precociously in all follicles, the timing of collective migration likely depends not only on changing ECM properties but also on cell receptor-extracellular ligand interactions.

(2) Cell-ECM interactions regulate the *speed* of epithelial sheet migration. Considering the equivalent amounts of basal integrins in S2–S8 FCs and assuming that the αPS1 βPS integrin affinity for laminin is similar during these stages, the fact that maximum speed is achieved precisely when laminin levels are highest indicates that laminin concentrations regulate migration speed. This hypothesis is reinforced by the demonstration that high Col IV levels in the BM are required for egg elongation (Isabella and Horne-Badovinac, 2015).

(3) The integrin-laminin balance plays a crucial role in the outcome of collective cell migration in vivo. Thus, the migration speed of FCs with decreased integrin amounts depends on the laminin concentration in the BM—the higher the laminin levels, the slower the rotation (i. e., *laminin* hypomorph *versus tj > mys RNAi*). Similarly, overexpression of integrins in laminin-depleted follicles can block migration.

(4) In the prevailing model of egg elongation, the polarized deposition of ECM fibrils is a consequence of follicle rotation and requires the polarized secretion of ECM components (Isabella and Horne-Badovinac, 2016). Furthermore, fibril deposition and a concomitant increase in tissue-level actin bundle alignment generate an instructive "molecular corset" that biases egg chamber growth toward the poles (Cetera et al., 2014). Consistent with this model, our results measuring

the mechanical properties of developing egg chambers show a marked increase in BM stiffness as rotation proceeds.

(5) Continuous, premature rotation in laminin-depleted follicles does not induce precocious BM polarization, indicating that rotation per se is not sufficient to generate a polarized ECM. It most likely also requires Rab10-dependent polarized FC fibril secretions containing Col IV, which, we propose, starts at S5, coinciding with a significant increase in Rab10 levels and with the initial decrease of perlecan amounts, assumed to facilitate the "constraining force" of the corset (Isabella and Horne-Badovinac, 2016; Lerner et al., 2013).

The establishment, stabilization, and disassembly of focal adhesions, the organization of the actin cytoskeleton, and the adhesion between migrating cells are factors known to reinforce migration and allow collective movement. Our results add to this knowledge and stress that key parameters, such as the initiation of collective migration and the speed of migration in vivo, can be controlled by variations in the levels of ECM ligands and cell receptors. Hence, when predicting the response of a cell collective to changes in the ECM, one ought to contemplate their levels of ECM receptors because increases in extracellular ligands can have opposite effects on cell behaviors depending on integrin levels. In our model, the ligand-receptor interactions that generate the traction forces required for movement are regulated during development, thus coordinating the timing of tissue growth and morphogenesis. However, our knowledge of the molecular details behind the initiation of collective cell migration is scarce. The findings reported here shed light on the cell biological mechanisms responsible for the initiation of coordinated movement. Considering that cell-ECM interactions are also a hallmark of physiological and pathological conditions that involve cell migration (such as tissue repair, cancer invasion, and immunity), the finding that collective migration is regulated by linked cellular and environmental properties broadens our understanding of the cellular basis of development and disease.

EXPERIMENTAL PROCEDURES

Fly Stocks

Flies were grown at 25°C on standard medium. *laminin* hypomorphs were *trans*-heterozygous for $LanB1^{28a}$ *(Urbano et al., 2009)* and *l(2)k05404* (Spradling et al., 1999), neither of which affect the coding region. *vkg* hypomorphs were *trans*-heterozygous for vkg^{01209} and vkg^{K07138} (Wang et al., 2008). The following fluorescence-tagged proteins were used: Fas3::GFP (Flybase P[PTT-GA] Fas3G00258; Flytrap G00258), Vkg::GFP (Flybase P[PTT-un1]vkgG205; Flytrap G205), and His2Av::mRFP (Flybase P[His2Av-mRFP1]). The *traffic jam-Gal4* driver *(tj-Gal4)* is expressed in the follicular epithelium and in the epithelial sheath (Andersen and Horne-Badovinac, 2016; Li et al., 2003). To knock down laminin, Abi, or integrin levels in the follicular epithelium, the following lines were used: *UAS-LanB1 RNAi* (VDRC 23119), *UAS-LanB2 RNAi* (VDRC 42559), *UAS-Abi RNAi* (DGRC-Kyoto 9749R), and *UAS-mys RNAi* (VDRC 29619). To overexpress the ßPS integrin subunit, we used the *UAS-ßPS* construct (Martin-Bermudo and Brown, 1996). Flies of the appropriate genotype were shifted from 18°C to 25°C for 3 days upon hatching and prior to dissection, except in the case of the *LanB1 RNAi* + *LanB2 RNAi* experiment, in which flies were shifted to 29°C.

Immunohistochemistry

Antibody, actin, and DNA stainings were performed following standard procedures. To analyze laminin, Col IV, and integrin levels, experimental and control ovaries were pooled and treated in parallel. Control ovaries carried the *ubi::GFP* or *His2Av::mRFP* markers (see experimental genotypes in the Supplemental Experimental Procedures). Images were acquired with exactly the same settings and quantified in parallel (see below; Figure S5).

Ex Vivo Ovariole Culture

Ovarioles were isolated from ovaries dissected in Schneider medium supplemented with 10% fetal bovine serum (Sigma, F3018), 0.6% (v/v) streptomycin/penicillin antibiotic mix (Invitrogen, 15140-122), and 0.20 mg ml^{-1} insulin (Sigma, 15500) as described previously (Valencia-Expósito et al., 2016). Individual ovarioles without the muscle sheath were transferred to a 35-mm poly-D-lysine-coated plate (Mattek, P35GC-1.5-10-C) containing supplemental Schneider medium and left to sink and settle in the bottom of the plate before image acquisition. Although culturing ovarioles ex vivo has been used extensively as a faithful method to study egg morphogenesis during oogenesis, we acknowledge that this is an experimental approach that may not completely reflect egg chamber maturation inside the female's abdomen.

Imaging of Fixed and Live Samples

Images were acquired with a Leica SP5 confocal microscope, analyzed utilizing Imaris and ImageJ, and processed with Adobe Photoshop and Adobe Illustrator. 3D images of fixed samples were taken with a 40×/1.3 numerical aperture (NA) oil immersion objective. 4D in vivo images were obtained at ~20°C. To analyze rotation, a 20×/0.7 NA oil immersion objective and Leica hybrid detectors (standard mode) were used, with time points every 2–4 min for 1–6 hr.

Transmission electron microscopy protocols and atomic force microscopy measurements are detailed in the Suppl. Information section.

Egg Shape Measurements

Maximum egg length and width of laid eggs from control and *laminin* hypomorph flies were used to calculate the aspect ratio (n = 100 for each genotype). Crosses were set with flies of the same age and grown at 25°C following standard procedures.

Data Analysis

The lineal velocity of FCs was calculated by manually tracking nuclei or geometrical cell centers using the Leica LAS AF software. Fluorescence quantification of control and experimental samples was performed on images captured using identical confocal settings (Figure S5). Quantification of actin bundle and Vkg::GFP fibril alignment was done using the "line ROI" tool of FIJI. See the Supplemental Experimental Procedures for further details.

Statistical Analysis

Experiments were performed with at least three biological replicas. Samples were collected from at least five different adult females grown under equivalent environmental conditions. The average values ± SD are represented (a.u.). p values were obtained using Student's t test to determine values that were significantly different ($p < 0.05$).

SUPPLEMENTAL INFORMATION

Supplemental Information includes Supplemental Experimental Procedures, five figures, three tables, and seven movies and can be found with this article online at http://dx.doi.org/10.1016/j.celrep.2017.06.031.

AUTHOR CONTRIBUTIONS

M.C.D.L., M.D.M.B., and A.G.R. conceived and designed the research. M.C.D.L., A.D.T., F.Z., A.E.R.N., E.M., M.D.M.B., and A.G.R. performed the research. M.C.D.L., A.D.T., F.Z., E.M., K.F., M.D.M.B., and A.G.R. analyzed the data. M.C.D.L., M.D.M.B., and A.G.R. wrote the paper.

ACKNOWLEDGMENTS

We thank the BDSC and the VDRC for fly stocks and the DSHB (University of Iowa) for antibodies. The technical support of Beatriz Ibáñez is acknowledged. TEM analysis was performed at the CIC, University of Granada. This work was funded by Spanish MINECO Grants BFU2015-65372 (to A.G.R.), BFU2013-48988-C2-01 (to M.D.M.B.), and Consolider CSD-2007-00008 (to M.D.M.B. and A.G.R.); by Junta de Andalucía Proyecto de Excelencia P09-CVI-5058 (to M.D.M.B. and A.G.R.); and by the European Regional Development Fund (FEDER). K.F. was supported by a Career Development Award from the UK Medical Research Council Grant G1100312/1. E.M. was supported by a Cambridge Herchel Smith Foundation fellowship. The help of Kathy García and Lesly Arbesú with image acquisition and analysis is acknowledged.

Received: February 14, 2017
Revised: May 26, 2017
Accepted: June 10, 2017
Published: July 5, 2017

REFERENCES

Andersen, D., and Horne-Badovinac, S. (2016). Influence of ovarian muscle contraction and oocyte growth on egg chamber elongation in Drosophila. Development *143*, 1375–1387.

Aurich, F., and Dahmann, C. (2016). A Mutation in fat2 Uncouples Tissue Elongation from Global Tissue Rotation. Cell Rep. *14*, 2503–2510.

Beck, K., Hunter, I., and Engel, J. (1990). Structure and function of laminin: anatomy of a multidomain glycoprotein. FASEB J. *4*, 148–160.

Bilder, D., and Haigo, S.L. (2012). Expanding the morphogenetic repertoire: perspectives from the Drosophila egg. Dev. Cell *22*, 12–23.

Bloch Qazi, M.C., Heifetz, Y., and Wolfner, M.F. (2003). The developments between gametogenesis and fertilization: ovulation and female sperm storage in Drosophila melanogaster. Dev. Biol. *256*, 195–211.

Cetera, M., Ramirez-San Juan, G.R., Oakes, P.W., Lewellyn, L., Fairchild, M.J., Tanentzapf, G., Gardel, M.L., and Horne-Badovinac, S. (2014). Epithelial rotation promotes the global alignment of contractile actin bundles during Drosophila egg chamber elongation. Nat. Commun. *5*, 5511.

Chen, D.Y., Lipari, K.R., Dehghan, Y., Streichan, S.J., and Bilder, D. (2016). Symmetry Breaking in an Edgeless Epithelium by Fat2-Regulated Microtubule Polarity. Cell Rep. *15*, 1125–1133.

Chi, H.C., and Hui, C.F. (1989). Primary structure of the Drosophila laminin B2 chain and comparison with human, mouse, and Drosophila laminin B1 and B2 chains. J. Biol. Chem. *264*, 1543–1550.

Devenport, D., and Brown, N.H. (2004). Morphogenesis in the absence of integrins: mutation of both Drosophila beta subunits prevents midgut migration. Development *131*, 5405–5415.

DiMilla, P.A., Barbee, K., and Lauffenburger, D.A. (1991). Mathematical model for the effects of adhesion and mechanics on cell migration speed. Biophys. J. *60*, 15–37.

Fernández-Miñán, A., Martín-Bermudo, M.D., and González-Reyes, A. (2007). Integrin signaling regulates spindle orientation in Drosophila to preserve the follicular-epithelium monolayer. Curr. Biol. *17*, 683–688.

Franze, K. (2011). Atomic force microscopy and its contribution to understanding the development of the nervous system. Curr. Opin. Genet. Dev. *21*, 530–537.

Friedl, P., and Gilmour, D. (2009). Collective cell migration in morphogenesis, regeneration and cancer. Nat. Rev. Mol. Cell Biol. *10*, 445–457.

Frydman, H.M., and Spradling, A.C. (2001). The receptor-like tyrosine phosphatase lar is required for epithelial planar polarity and for axis determination within *drosophila* ovarian follicles. Development *128*, 3209–3220.

Galbraith, C.G., Yamada, K.M., and Galbraith, J.A. (2007). Polymerizing actin fibers position integrins primed to probe for adhesion sites. Science *315*, 992–995.

Gates, J. (2012). Drosophila egg chamber elongation: insights into how tissues and organs are shaped. Fly (Austin) *6*, 213–227.

González-Reyes, A., and St Johnston, D. (1998). The *Drosophila* AP axis is polarised by the cadherin-mediated positioning of the oocyte. Development *125*, 3635–3644.

González-Reyes, A., Elliott, H., and St Johnston, D. (1997). Oocyte determination and the origin of polarity in Drosophila: the role of the spindle genes. Development *124*, 4927–4937.

Graner, M.W., Bunch, T.A., Baumgartner, S., Kerschen, A., and Brower, D.L. (1998). Splice variants of the Drosophila PS2 integrins differentially interact with RGD-containing fragments of the extracellular proteins tiggrin, ten-m, and D-laminin 2. J. Biol. Chem. *273*, 18235–18241.

Gupton, S.L., and Waterman-Storer, C.M. (2006). Spatiotemporal feedback between actomyosin and focal-adhesion systems optimizes rapid cell migration. Cell *125*, 1361–1374.

Gutzeit, H.O., Eberhardt, W., and Gratwohl, E. (1991). Laminin and basement membrane-associated microfilaments in wild-type and mutant Drosophila ovarian follicles. J. Cell Sci. *100*, 781–788.

Haigo, S.L., and Bilder, D. (2011). Global tissue revolutions in a morphogenetic movement controlling elongation. Science *331*, 1071–1074.

Harunaga, J.S., Doyle, A.D., and Yamada, K.M. (2014). Local and global dynamics of the basement membrane during branching morphogenesis require protease activity and actomyosin contractility. Dev. Biol. *394*, 197–205.

Henchcliffe, C., García-Alonso, L., Tang, J., and Goodman, C.S. (1993). Genetic analysis of laminin A reveals diverse functions during morphogenesis in *Drosophila*. Development *118*, 325–337.

Hohenester, E., and Yurchenco, P.D. (2013). Laminins in basement membrane assembly. Cell Adhes. Migr. *7*, 56–63.

Horne-Badovinac, S., Hill, J., Gerlach, G., 2nd, Menegas, W., and Bilder, D. (2012). A screen for round egg mutants in Drosophila identifies tricornered, furry, and misshapen as regulators of egg chamber elongation. G3 (Bethesda) *2*, 371–378.

Huang, C., Rajfur, Z., Borchers, C., Schaller, M.D., and Jacobson, K. (2003). JNK phosphorylates paxillin and regulates cell migration. Nature *424*, 219–223.

Huttenlocher, A., Ginsberg, M.H., and Horwitz, A.F. (1996). Modulation of cell migration by integrin-mediated cytoskeletal linkages and ligand-binding affinity. J. Cell Biol. *134*, 1551–1562.

Hynes, R.O. (1992). Integrins: versatility, modulation, and signaling in cell adhesion. Cell *69*, 11–25.

Isabella, A.J., and Horne-Badovinac, S. (2015). Dynamic regulation of basement membrane protein levels promotes egg chamber elongation in Drosophila. Dev. Biol. *406*, 212–221.

Isabella, A.J., and Horne-Badovinac, S. (2016). Rab10-Mediated Secretion Synergizes with Tissue Movement to Build a Polarized Basement Membrane Architecture for Organ Morphogenesis. Dev. Cell *38*, 47–60.

Kumagai, C., Kadowaki, T., and Kitagawa, Y. (1997). Disulfide-bonding between Drosophila laminin β and γ chains is essential for α chain to form α betagamma trimer. FEBS Lett. *412*, 211–216.

Kusche-Gullberg, M., Garrison, K., MacKrell, A.J., Fessler, L.I., and Fessler, J.H. (1992). Laminin A chain: expression during Drosophila development and genomic sequence. EMBO J. *11*, 4519–4527.

Lerner, D.W., McCoy, D., Isabella, A.J., Mahowald, A.P., Gerlach, G.F., Chaudhry, T.A., and Horne-Badovinac, S. (2013). A Rab10-dependent mechanism for polarized basement membrane secretion during organ morphogenesis. Dev. Cell *24*, 159–168.

Lewellyn, L., Cetera, M., and Horne-Badovinac, S. (2013). Misshapen decreases integrin levels to promote epithelial motility and planar polarity in Drosophila. J. Cell Biol. *200*, 721–729.

Li, M.A., Alls, J.D., Avancini, R.M., Koo, K., and Godt, D. (2003). The large Maf factor Traffic Jam controls gonad morphogenesis in *Drosophila*. Nat. Cell Biol. *5*, 994–1000.

Lo, C.M., Wang, H.B., Dembo, M., and Wang, Y.L. (2000). Cell movement is guided by the rigidity of the substrate. Biophys. J. *79*, 144–152.

Lunstrum, G.P., Bächinger, H.P., Fessler, L.I., Duncan, K.G., Nelson, R.E., and Fessler, J.H. (1988). Drosophila basement membrane procollagen IV. I. Protein characterization and distribution. J. Biol. Chem. *263*, 18318–18327.

Markow, T.A., Beall, S., and Matzkin, L.M. (2009). Egg size, embryonic development time and ovoviviparity in Drosophila species. J. Evol. Biol. *22*, 430–434.

Martin, D., Zusman, S., Li, X., Williams, E.L., Khare, N., DaRocha, S., Chiquet-Ehrismann, R., and Baumgartner, S. (1999). wing blister, a new *Drosophila* laminin alpha chain required for cell adhesion and migration during embryonic and imaginal development. J. Cell Biol. *145*, 191–201.

Martin-Bermudo, M.D., and Brown, N.H. (1996). Intracellular signals direct integrin localization to sites of function in embryonic muscles. J. Cell Biol. *134*, 217–226.

Montell, D.J., and Goodman, C.S. (1989). Drosophila laminin: sequence of B2 subunit and expression of all three subunits during embryogenesis. J. Cell Biol. *109*, 2441–2453.

Morrissey, M.A., and Sherwood, D.R. (2015). An active role for basement membrane assembly and modification in tissue sculpting. J. Cell Sci. *128*, 1661–1668.

O'Reilly, A.M., Lee, H.H., and Simon, M.A. (2008). Integrins control the positioning and proliferation of follicle stem cells in the Drosophila ovary. J. Cell Biol. *182*, 801–815.

Palecek, S.P., Loftus, J.C., Ginsberg, M.H., Lauffenburger, D.A., and Horwitz, A.F. (1997). Integrin-ligand binding properties govern cell migration speed through cell-substratum adhesiveness. Nature *385*, 537–540.

Pastor-Pareja, J.C., and Xu, T. (2011). Shaping cells and organs in Drosophila by opposing roles of fat body-secreted Collagen IV and perlecan. Dev. Cell *21*, 245–256.

Rasmussen, J.P., Reddy, S.S., and Priess, J.R. (2012). Laminin is required to orient epithelial polarity in the C. elegans pharynx. Development *139*, 2050–2060.

Reig, G., Pulgar, E., and Concha, M.L. (2014). Cell migration: from tissue culture to embryos. Development *141*, 1999–2013.

Schneider, M., Khalil, A.A., Poulton, J., Castillejo-Lopez, C., Egger-Adam, D., Wodarz, A., Deng, W.M., and Baumgartner, S. (2006). Perlecan and Dystroglycan act at the basal side of the Drosophila follicular epithelium to maintain epithelial organization. Development *133*, 3805–3815.

Spradling, A. (1993). Developmental genetics of oogenesis. In The Development of Drosophila melanogaster, M. Bate and A. Martinez-Arias, eds. (New York: Cold Spring Harbor Laboratory press), pp. 1–70.

Spradling, A.C., Stern, D., Beaton, A., Rhem, E.J., Laverty, T., Mozden, N., Misra, S., and Rubin, G.M. (1999). The Berkeley Drosophila Genome Project gene disruption project: Single P-element insertions mutating 25% of vital Drosophila genes. Genetics *153*, 135–177.

Timpl, R., Rohde, H., Robey, P.G., Rennard, S.I., Foidart, J.M., and Martin, G.R. (1979). Laminin–a glycoprotein from basement membranes. J. Biol. Chem. *254*, 9933–9937.

Urbano, J.M., Torgler, C.N., Molnar, C., Tepass, U., López-Varea, A., Brown, N.H., de Celis, J.F., and Martín-Bermudo, M.D. (2009). Drosophila laminins act as key regulators of basement membrane assembly and morphogenesis. Development *136*, 4165–4176.

Valencia-Expósito, A., Grosheva, I., Míguez, D.G., González-Reyes, A., and Martín-Bermudo, M.D. (2016). Myosin light-chain phosphatase regulates basal actomyosin oscillations during morphogenesis. Nat. Commun. *7*, 10746.

Viktorinová, I., and Dahmann, C. (2013). Microtubule polarity predicts direction of egg chamber rotation in Drosophila. Curr. Biol. *23*, 1472–1477.

Wang, X., Harris, R.E., Bayston, L.J., and Ashe, H.L. (2008). Type IV collagens regulate BMP signalling in Drosophila. Nature *455*, 72–77.

Wehrle-Haller, B. (2012). Assembly and disassembly of cell matrix adhesions. Curr. Opin. Cell Biol. *24*, 569–581.

Wickström, S.A., Radovanac, K., and Fässler, R. (2011). Genetic analyses of integrin signaling. Cold Spring Harb. Perspect. Biol. *3*, a005116.

Woodruff, R.C., and Ashburner, M. (1979). The genetics of a small autosomal region of *Drosophila* melanogaster containing the structural gene for alcohol dehydrogenase. II. Lethal mutations in the region. Genetics *92*, 133–149.

Yurchenco, P.D. (2011). Basement membranes: cell scaffoldings and signaling platforms. Cold Spring Harb. Perspect. Biol. *3*, a004911++.

Zaidel-Bar, R., and Geiger, B. (2010). The switchable integrin adhesome. J. Cell Sci. *123*, 1385–1388.

Cell Reports, Volume *20*

Supplemental Information

Laminin Levels Regulate Tissue Migration and Anterior-Posterior Polarity during Egg Morphogenesis in *Drosophila*

María C. Díaz de la Loza, Alfonsa Díaz-Torres, Federico Zurita, Alicia E. Rosales-Nieves, Emad Moeendarbary, Kristian Franze, María D. Martín-Bermudo, and Acaimo González-Reyes

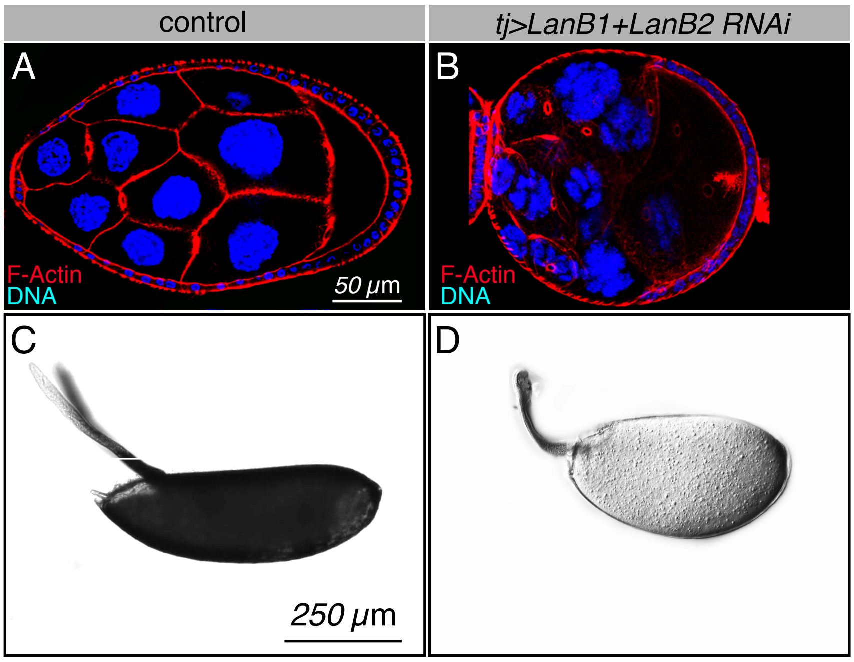

Supplemental Figure 1

Supplemental Figure 1 (related to Figures 1 and 2): Strong laminin depletion blocks egg elongation and follicle migration. **(A)** S5-S7 wild-type egg chambers are elongated along the anterior-posterior axis. **(B)** Strong depletion of laminin levels in *tj>LanB1+LanB2 RNAi* ovaries prevents egg elongation. Thus, S5-S7 egg chambers appear rounded (100% of cases, n=54). **(C)** The large majority of eggs laid by control females are properly elongated (number of eggs analysed (n)=178; length/width ratio ~2.75). **(D)** *tj>LanB1+LanB2 RNAi* females lay 86% eggs rounder than controls (n=89; length/width ratio <2.5). Filamentous actin was detected with rhodamine-labelled phalloidin.

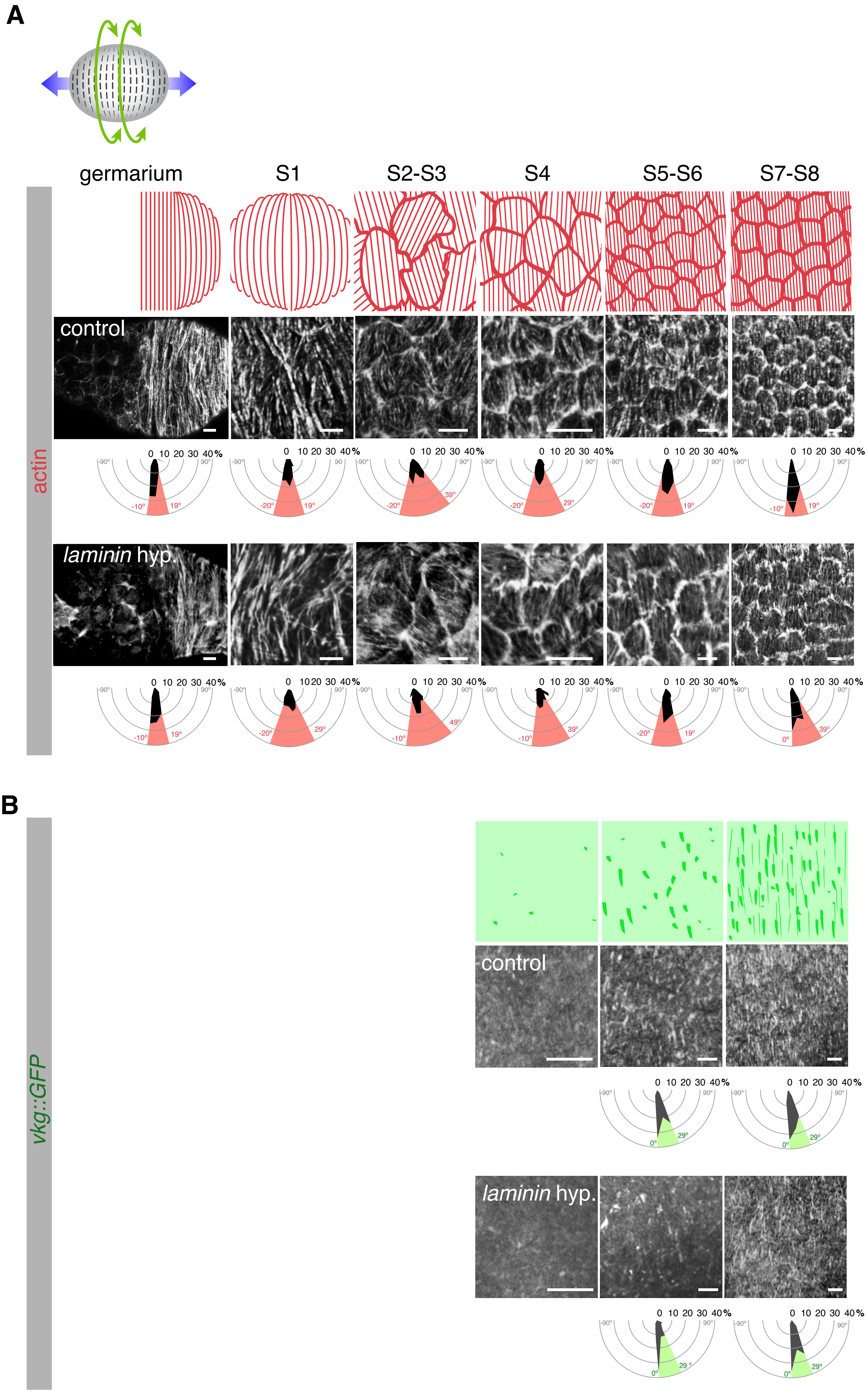

Supplemental Figure 2

Supplemental Figure 2 (related to Figure 2): Basal planar cell polarity of follicle cells and organisation of Col IV fibrils is not affected in *laminin* hypomorphic ovarioles. (A) Schematic representation of the distribution of basal actin filaments in follicle cells during egg chamber development (top). Visualisation of filamentous actin with rhodamine-labelled phalloidin in control (middle) and *laminin* hypomorphic ovarioles (bottom). Actin filaments are clearly organised perpendicular to the anterior-posterior axis of the ovariole in germarial stages and from S5 to S8. S2-S4 egg chambers display a less obvious polarisation. Radial diagrams indicate the percentage of bundles found within a given sector. The black areas correspond to the data reported in Table S2. Angles delimiting the sectors (depicted in pink) are shown. **(B)** Schematic representation of the organisation of the basement membrane component Col IV (top). Immunodetection of the protein trap Vkg::GFP in control (middle) and *laminin* hypomorphic ovarioles (bottom) show that Collagen IV is polarised perpendicular to the ovariole's AP axis from S5. Radial diagrams indicate the percentage of fibrils found within a given sector. The black areas correspond to the data reported in Table S4. Scale bars = 5 microns.

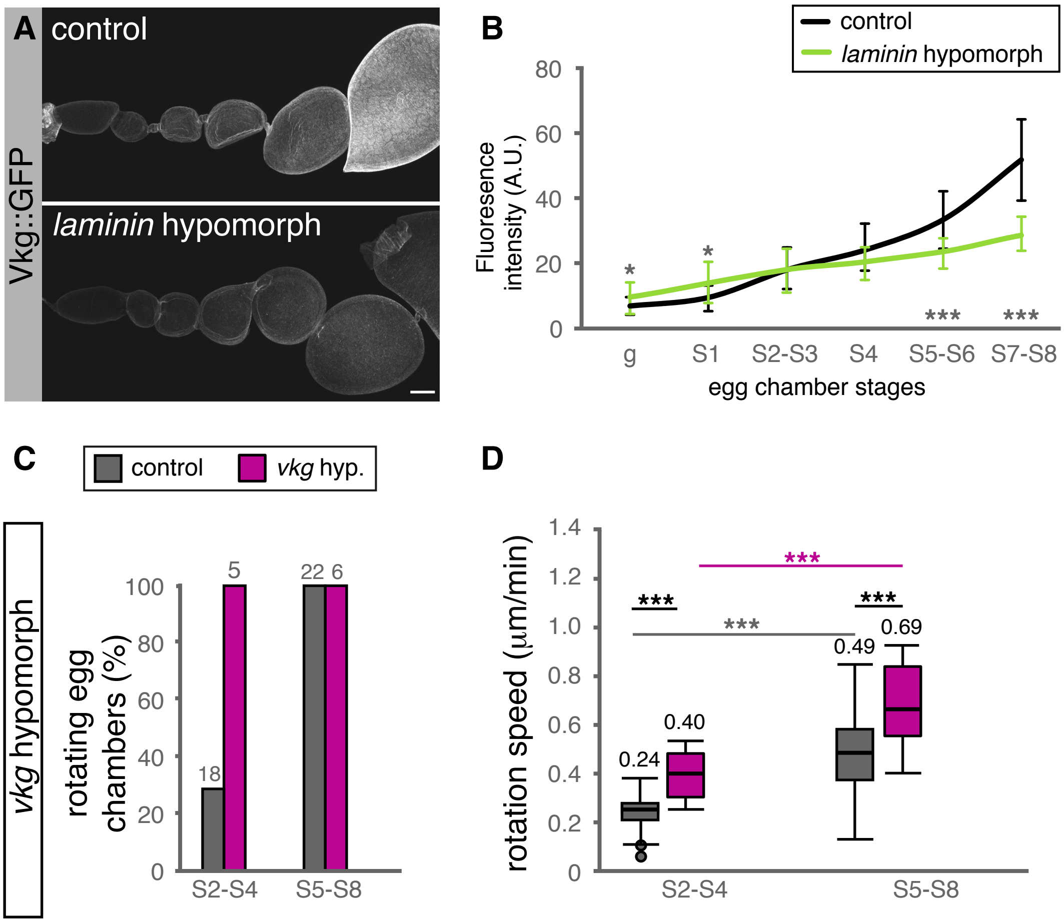

Supplemental Figure 3

Supplemental Figure 3 (related to Figure 2): Col IV deposition is affected in S7-8 *laminin* hypomorphs. Col IV is required for proper rotation. (A) Immunodetection of Col IV by means of the protein trap Vkg::GFP in control and *laminin* hypomorphic ovarioles using an anti-GFP antibody. Image is a maximum projection of at least 15 sections along the Z-axis. **(B)** Quantification of the immunofluorescence signal in different egg chambers is shown. Mean and standard deviation of measurements in 3 to 5 ovarioles are indicated. **(C)** Onset of rotation and **(D)** quantification of rotation speed of *vkg* hypomorphic follicles at different stages. Horizontal lines in boxes represent MEDIAN values of a minimum of 11 and a maximum of 103 measurements per genotype and stage. Values above boxes indicate MEAN rotation speeds. See Movies S1 and S7. P values of two-tailed t-tests <0.05 were considered statistically significant (*: P<0.05, **: P<0.005, ***: P<0.001). Scale bar = 25 microns.

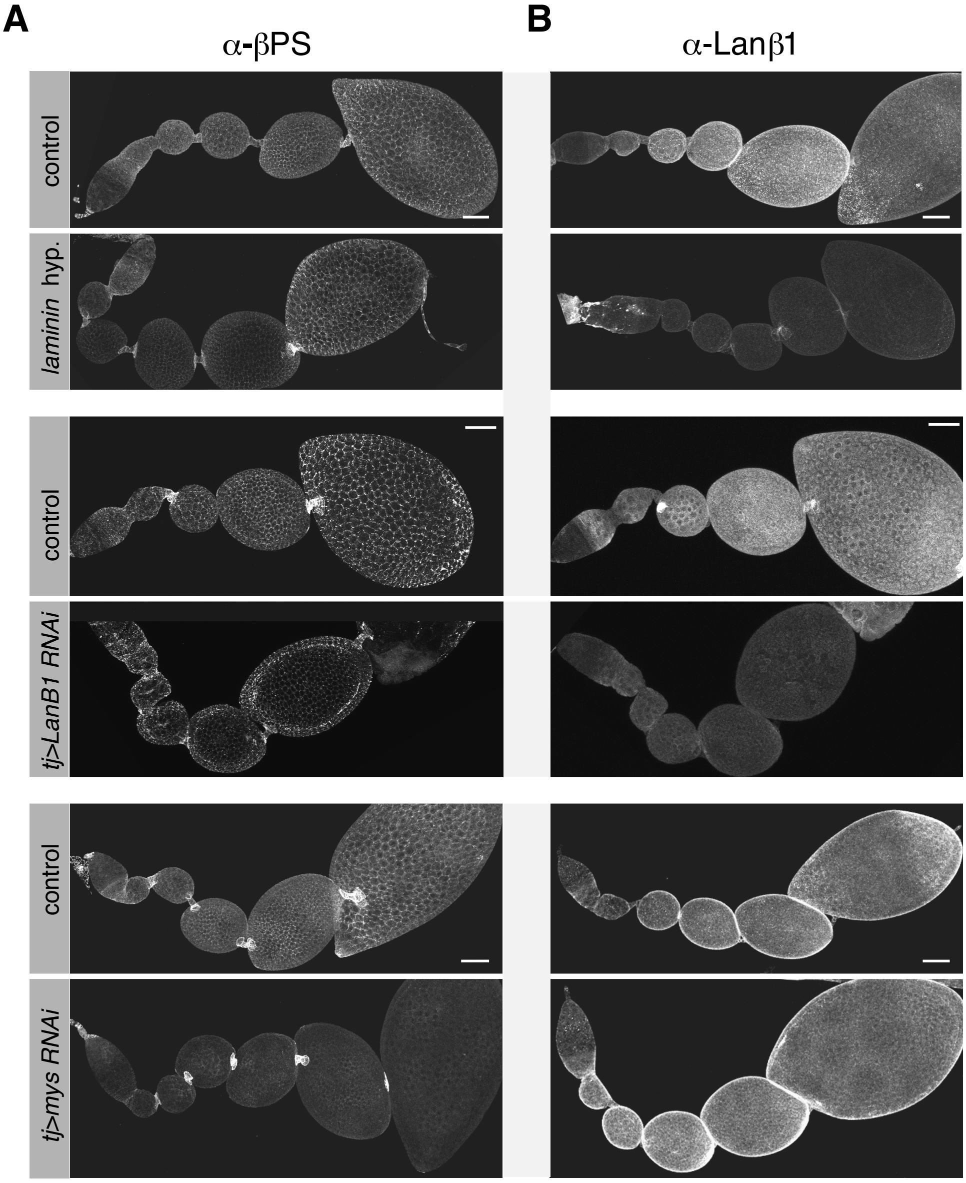

Supplemental Figure 4

Supplemental Figure 4 (related to Figure 4): **Visualisation of ßPS integrin and Laminin ß1 in control and experimental ovarioles. (A)** Immunodetection of ßPS and **(B)** Laminin ß1 during oogenesis in controls and in different genetic backgrounds. See Supplementary Table 1 for the quantification of fluorescence data. Images are maximum projections of at least 15 sections along the Z axis. Scale bars = 25 microns.

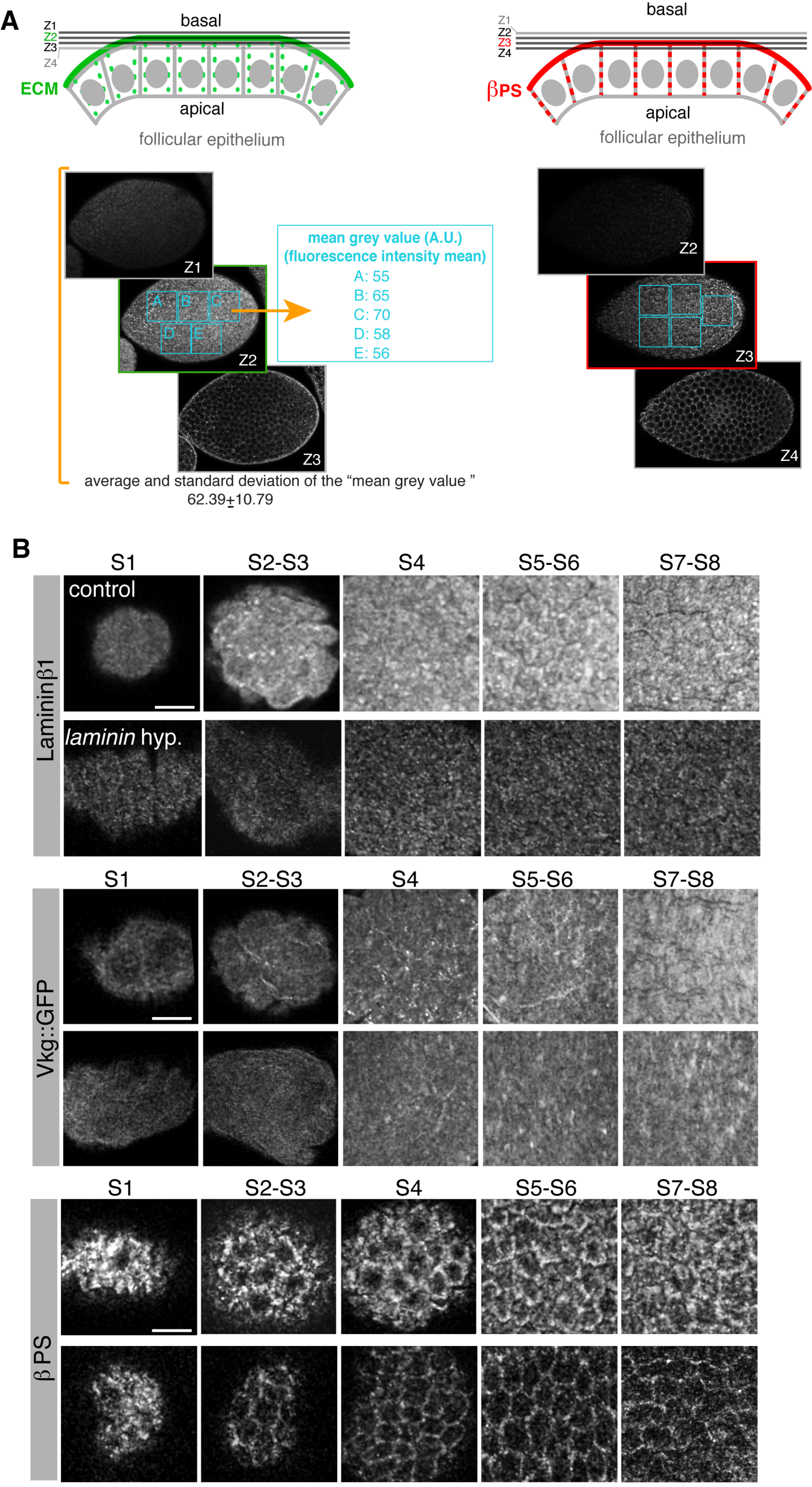

Supplemental Figure 5

Supplemental Figure 5 (related to Figures 1, 4, S3 and S4): **Quantification of immunofluorescence of basement membrane components and basal integrin**. **(A)** Schematic representation of the focal planes used to quantify immunofluorescence levels of BM components (green, left) and basal integrin (red, right). Confocal sections were taken through the egg chamber at 0.8-1.0 μm intervals (Z1, Z2, Z3 and Z4). Z2 cut through the plane of the BM (green line; to quantify ECM components). Membrane or cytoplasmic signals (green dashed lines) were discarded in BM measurements. Z3 focused on the basal side of the follicle cells (red line; to quantify integrin levels). Fluorescent signal from the lateral membrane of follicle cells (red dashed lines) was avoided in integrin measurements. For a given confocal section, five non-overlaping flat areas (cyan squares) were chosen to avoid edge effects and their mean "grey value" was calculated for each area using Imaris and/or ImageJ software (as shown for the BM example in **A**; see Suppl. Experimental Procedures for further details). Average fluorescence intensities ± standard deviations were calculated from ≥3 egg chambers of the appropriate stage (i. e., at least 15 measurements per stage). **(B)** Examples of high magnification images of basal laminin, Col IV and integrin immunofluorescence used for the analyses of protein levels in both control and experimental S1-S8 egg chambers. Scale bar = 5 microns.

SUPPLEMENTAL TABLE 1 (related to Figs. 1, 2, 4, S3 and S4)

Quantification of the immunofluorescence signal (A.U.) of Vkg::GFP, α-βPS and α-Laminin β1, and of rotation speed during oogenesis

**genotype**	**egg chamber stage**					
	**germarium**	**S1**	**S2-S3**	**S4**	**S5-S6**	**S7-S8**
**collagen IV levels**						
control	**6.10**±2.41	**9.15**±4.46	**17.91**±7.00	**24.05**±7.07	**33.34**±8.82	**52.00**±13.02
laminin hypomorph	**8.86**±4.72	**13.77**±6.19	**18.12**±6.29	**20.00**±5.59	**22.90**±4.70	**28.50**±5.26
fold change	*1.45*	*1.50*	*1.01*	*0.83*	*0.67*	*0.55*
	*	*			***	***
**laminin levels**						
control	**21.66**±3.63	**41.01**±15.3	**48.78**±12.7	**67.91**±5.84	**67.42**±21.86	**62.39**±10.79
laminin hypomorph	**22.82**±3.12	**21.53**±4.31	**19.51**±1.63	**20.58**±3.66	**17.66**±2.13	**16.60**±2.80
fold change	*1.05*	*0.53*	*0.40*	*0.31*	*0.26*	*0.27*
		***	***	***	***	***
control	**21.81**±3.63	**40.42**±13.6	**46.53**±11.3	**59.78**±15.31	**66.98**±18.74	**60.57**±13.40
tj>LanB1 RNAi	**13.65**±4.32	**21.82**±7.62	**31.03**±4.00	**28.31**±9.06	**27.54**±9.18	**28.76**±8.02
fold change	*0.63*	*0.54*	*0.67*	*0.47*	*0.41*	*0.47*
	***	***	***	***	***	***
control	**27.20**±2.05	**43.29**±9.58	**46.01**±11	**53.83**±5.65	**61.55**±9.09	**57.42**±15.42
tj>mys RNAi	**28.21**±4.48	**69.49**±7.81	**73.54**±12.7	**74.04**±8.33	**67.62**±14.91	**56.08**±16.67
fold change	*1.04*	*1.61*	*1.60*	*1.38*	*1.10*	*0.98*
		***	**	***		
**integrin levels**						
control	**12.00**±5.42	**42.24**±4.40	**36.93**±2.26	**34.79**±3.52	**38.15**±4.02	**38.32**±5.16
laminin hypomorph	**12.20**±2.86	**30.77**±5.49	**18.75**±5.17	**14.77**±4.77	**18.88**±4.19	**21.30**±5.70
fold change	*1.02*	*0.73*	*0.51*	*0.42*	*0.49*	*0.56*
		***	***	***	***	***
control	**14.53**±6.27	**44.44**±13.1	**36.86**±9.98	**38.55**±8.42	**39.85**±6.57	**39.39**±7.52
tj>LanB1 RNAi	**13.71**±3.91	**26.48**±10.9	**26.76**±8.77	**27.98**±9.33	**28.34**±8.21	**27.95**±3.78
fold change	*0.69*	*0.60*	*0.73*	*0.76*	*0.78*	*0.78*
		***	***	***	***	***
control	**19.20**±3.11	**43.10**±8.31	**34.54**±5.67	**34.22**±4.77	**38.06**±5.09	**38.20**±9.63
tj>mys RNAi	**20.72**±5.16	**12.90**±2.96	**18.41**±4.30	**22.18**±3.81	**24.02**±5.33	**19.88**±2.45
fold change	*1.08*	*0.30*	*0.53*	*0.65*	*0.63*	*0.52*
		***	***	***	***	***
**Rotation speed (μm/min)**			**S2-S4**		**S5-S8**	
control			**0.24**±0.08		**0.49**±0.17	
laminin hypomorph			**0.56**±0.18		**0.62**±0.27	
tj>LanB1 RNAi			**0.50**±0.09		**0.79**±0.16	
viking hypomorph			**0.40**±0.10		**0.69**±0.16	
tj>mys RNAi			**0.21**±0.06		**0.40**±0.16	
tj>βPS			**0.37**±0.17		**0.65**±0.26	

The fold change in fluorescence levels between mutant and control egg chambers is shown. Mean and standard deviation of a minimum of 11 and a maximum of 103 measurements per genotype and stage are indicated. See Supplementary Fig. 4 for examples of the original data. P values <0.05 were considered statistically significant (*:P<0.05, **:P<0.005, ***:P<0.001).

Supplemental Table 2 (related to Fig. S2)

genotype	angle (°)		Actin bundle alignment with respect to the AP axis (%)					
			egg chamber stage					
			germarium	S1	S2-S3	S4	S5-S6	S7-S8
**control**								
	-90	-81	*2.96*	*0*	*0*	*0*	*0*	*0*
	-80	-71	*1.48*	*0.57*	*0*	*0*	*0*	*0*
	-70	-61	*0.00*	*1.14*	*1.61*	*0.56*	*0*	*0*
	-60	-51	*0.00*	*2.84*	*1.08*	*1.12*	*0*	*0*
	-50	-41	*0.00*	*1.70*	*1.08*	*2.25*	*0*	*1.61*
	-40	-31	*2.22*	*3.41*	*3.76*	*2.25*	*0.83*	*1.61*
	-30	-21	*3.70*	*7.39*	*5.38*	*6.74*	*6.67*	*0.54*
	-20	-11	*8.15*	*15.91*	*11.83*	*10.11*	*12.50*	*2.69*
	-10	1	*26.67*	*15.34*	*17.74*	*14.04*	*22.50*	*27.42*
	0	9	*26.67*	*19.32*	*9.68*	*18.54*	*25.00*	*39.25*
	10	19	*10.37*	*12.50*	*13.98*	*14.61*	*16.67*	*21.51*
	20	29	*5.19*	*5.68*	*15.59*	*10.11*	*5.83*	*4.30*
	30	39	*1.48*	*6.82*	*11.83*	*6.18*	*4.17*	*1.08*
	40	49	*0.74*	*2.84*	*4.84*	*5.62*	*0.83*	*1.61*
	50	59	*1.48*	*2.27*	*1.08*	*3.37*	*0.83*	*1.61*
	60	69	*8.89*	*1.70*	*0.54*	*2.25*	*2.50*	*0*
	70	79	*0.74*	*0.57*	*0*	*1.12*	*0.83*	*0*
	80	90	*0*	*0*	*0*	*0.56*	*0.83*	*0*
***laminin* hypomorph**								
	-90	-81	*0*	*1.19*	*0.58*	*0*	*0*	*0*
	-80	-71	*0*	*1.19*	*0.58*	*0.73*	*0*	*0*
	-70	-61	*0*	*8.33*	*0.58*	*2.92*	*0*	*0.60*
	-60	-51	*0*	*1.19*	*1.16*	*2.92*	*0*	*0.60*
	-50	-41	*1.12*	*1.19*	*1.16*	*2.19*	*0.58*	*0.91*
	-40	-31	*4.49*	*7.14*	*1.16*	*2.19*	*1.16*	*0.91*
	-30	-21	*5.62*	*4.76*	*2.89*	*5.11*	*5.78*	*0.91*
	-20	-11	*8.99*	*8.33*	*6.36*	*5.11*	*9.83*	*3.63*
	-10	1	*19.10*	*17.86*	*11.56*	*10.22*	*23.70*	*5.14*
	0	9	*24.72*	*17.86*	*11.56*	*13.14*	*20.23*	*29.61*
	10	19	*25.84*	*10.71*	*12.14*	*12.41*	*17.92*	*20.85*
	20	29	*7.87*	*10.71*	*15.61*	*8.03*	*6.36*	*23.26*
	30	39	*2.25*	*8.33*	*13.87*	*9.49*	*6.36*	*9.67*
	40	49	*0*	*1.19*	*10.40*	*2.92*	*4.05*	*2.42*
	50	59	*0*	*0*	*4.62*	*8.76*	*1.73*	*1.21*
	60	69	*0*	*0*	*2.31*	*7.30*	*0.58*	*0*
	70	79	*0*	*0*	*2.31*	*2.19*	*1.16*	*0*
	80	90	*0*	*0*	*0*	*2.19*	*0.58*	*0.30*

Quantification of the relative angles of basal actin bundles of follicle cells. A 0° angle represents a bundle perpendicular to the AP axis. Values refer to the percentage of bundles with a given orientation with respect to the AP axis of the ovariole. Intervals that contain at least 9.5% of the scored bundles are highlighted in red. A minimum of 100 bundles were analysed from at least 5 egg chambers per stage.

SUPPLEMENTAL TABLE 3 (related to Fig. S2)

Col IV fibril alignment with respect to the AP axis (%)

genotype	angle (°)		Egg chamber stage	
			S5-S6	S7-S8
**control**				
	-90	-81	*0*	*0*
	-80	-71	*0*	*0*
	-70	-61	*0*	*0*
	-60	-51	*0*	*0*
	-50	-41	*0*	*0*
	-40	-31	*0*	*1.44*
	-30	-21	*1.10*	*1.44*
	-20	-11	*4.40*	*2.01*
	-10	1	*7.69*	*8.91*
	0	9	*32.97*	*34.48*
	10	19	*19.78*	*27.30*
	20	29	*24.18*	*19.25*
	30	39	*5.49*	*4.89*
	40	49	*3.30*	*0.29*
	50	59	*1.10*	*0*
	60	69	*0*	*0*
	70	79	*0*	*0*
	80	90	*0*	*0*
***laminin* hypomorph**				
	-90	-81	*0*	*0*
	-80	-71	*0*	*0*
	-70	-61	*0*	*0*
	-60	-51	*0*	*0*
	-50	-41	*0*	*0.58*
	-40	-31	*0*	*0.58*
	-30	-21	*3.90*	*0.58*
	-20	-11	*2.60*	*2.89*
	-10	1	*6.49*	*5.20*
	0	9	*36.36*	*36.42*
	10	19	*12.99*	*20.81*
	20	29	*12.99*	*24.86*
	30	39	*7.79*	*8.09*
	40	49	*5.19*	*0*
	50	59	*3.90*	
	60	69	*3.90*	*0*
	70	79	*3.90*	*0*
	80	90	*0*	*0*

Quantification of the relative angles of Collagen IV:GFP fibrils. A 0° angle represents a fibril perpendicular to the AP axis. Values refer to the percentage of fibrils with a given orientation with respect to the AP axis of the ovariole. Intervals that contain at least 10% of the scored bundles are highlighted in green. A minimum of 50 fibrils were analysed from at least 5 egg chambers per stage.

Supplemental Experimental Procedures

Experimental genotypes

Figure 1
control: *w*; *ubi-GFP FRT40/CyO*
laminin hypomorph: *w/w; l(2)k05404/LanB1*^{28a}
tj>LanB1 RNAi: UAS-dicer/+; tj-Gal4/+; UAS-LanB1 RNAi/tub-Gal80^{ts}

Suppl. Figure 1
tj>LanB1+LanB2 RNAi: UAS-dicer/+; tj-Gal4/UAS-LanB2 RNAi; UAS-LanB1 RNAi/tub-Gal80^{ts}

Figure 2; Suppl. Movies S1 and S2
control: *w*; *Fas3::GFP/+*; *His2Av::mRFP/+*
laminin hypomorph: *w*; *l(2)k05404 Fas3::GFP/LanB1*^{28a} *His2Av::mRFP*
tj>LanB1 RNAi: UAS-dicer/+; tj-Gal4/+; UAS-LanB1 RNAi/tub-Gal80^{ts}

Suppl. Figures S2 and S3
control: *w*; *l(2)k05404, vkg::GFP/+; +/TM2*
laminin hypomorph: *w*; *l(2)k05404, vkg::GFP/LanB1*^{28a} *His2Av::mRFP*
viking hypomorph: y *w/+*; *vkg*^{01209}/ *vkg*^{K07138}*; ry[506]/+*

Figure 3
control: *w*; *ubi-GFP FRT40/CyO*
laminin hypomorph: *w/w; l(2)k05404/LanB1*^{28a}

Figure 4; Suppl. Figure 4; Suppl. Movie S2
control: *w*; *ubi-GFP FRT40/CyO*
control: *w*; *Fas3::GFP/+*; *His2Av::mRFP/+*
laminin hypomorph: *w/w; l(2)k05404/LanB1*^{28a}
laminin hypomorph: *w*; *(l)2k05404 Fas3::GFP/LanB1*^{28a} *His2Av::mRFP*
tj>LanB1 RNAi: UAS-dicer/+; tj-Gal4/+; UAS-LanB1 RNAi/tub-Gal80^{ts}
tj>mys RNAi: w; UAS-mys RNAi/tj-Gal4; tub-Gal80^{ts}*/+*
tj>ßPS: UAS-ßPS/ traffic jam-Gal4; tub-Gal80^{ts}*/+*
tj>LanB1 RNAi+ßPS (control): UAS-dicer/+; tj-Gal4/UAS-GFP; UAS-LanB1 RNAi/tub-Gal80^{ts}
tj>LanB1 RNAi+ßPS: UAS-dicer/+; tj-Gal4/UAS-ßPS; UAS-LanB1 RNAi/tub-Gal80^{ts}

Figure 5
control: *w*; *ubi-GFP FRT40/CyO*
laminin hypomorph: *w/w; l(2)k05404/LanB1*^{28a}
tj>LanB1 RNAi: UAS-dicer/+; tj-Gal4/+; UAS-LanB1 RNAi/tub-Gal80^{ts}

Figure 6; Suppl. Movies S4-6
control: *w*; *Fas3::GFP/+*; *His2Av::mRFP/+*
laminin hypomorph: *w*; *l(2)k05404 Fas3::GFP/LanB1*^{28a} *His2Av::mRFP*
tj>lanB1 RNAi+GFP (control): UAS-dicer/+; tj-Gal4/tub-Gal80^{ts}*; UAS-LanB1 RNAi/UAS-GFP*
tj>lanB1 RNAi+Abi RNAi: UAS-dicer/+; tj-Gal4/tub-Gal80^{ts}*; UAS-LanB1 RNAi/UAS-Abi RNAi*

Figure 7; Suppl. Movie S7
control: *w*; *Fas3::GFP/+*; *His2Av::mRFP/+*
laminin hypomorph: *w*; *l(2)k05404 Fas3::GFP/LanB1*^{28a} *His2Av::mRFP*
spindle mutant egg chamber from a homozygous female: *w;; ru spn-C*^{094} *e spn-A*^{057} *ca*

Immunohistochemistry

Primary antibodies were used at the following concentrations: rabbit anti-Laminin b1 (Kumagai et al., 1997), 1:1000; FITC-conjugated anti-GFP (Abcam, ab6662), 1:500; anti-Fasciclin3 mouse monoclonal, 1:50 (7G10 anti-Fasciclin III was deposited to the DSHB by Goodman, C.); anti-orb mouse monoclonal, 1:10 (a mixture of Orb 4H8 and Orb 6H4 monoclonal antibodies, 1:20 each; 4H8 and 6H4 anti-Orb were deposited to the DSHB by

Schedl, P.); anti-bPS mouse monoclonal, 1:100 (CF.6G11 anti-βPS was deposited to the DSHB by Brower, D.).

Imaging of ovarian tissues
3-D images of fixed samples were acquired using a 40x/1.3 NA oil immersion objective. 4-D *in vivo* images were acquired at room temperature (~20°C). We used a Leica SP5 confocal microscope controlled by the Leica LAS AF software. To analyse rotation, we performed live imaging of complete ovarioles using a 20x/0.7 NA oil immersion objective and Leica hybrid detectors (standard mode), with time points every 2-4 minutes for 1-6 hours. Follicle cell migration was analysed either with the Fas3::GFP and His2Av::mRFP fluorescence proteins or with transmitted light (phase contrast). Rendering of 4-D live images were performed in Imaris. Snapshots were obtained and processed using ImageJ and Adobe Photoshop, and labelled in Adobe Illustrator.

Transmission Electron Microscopy (TEM)
Ovaries were dissected in Phosphate Buffer Saline + 0.1% Tween-20 and fixed for 2 hours at 4 ^{0}C in 3% glutaraldehyde/l% paraformaldehyde (vol./vol.) in 0.05 M cacodylate buffer (pH 7.4). After three 10 min. washes in cacodylate buffer 0.1 M at 4 ^{0}C, ovaries were postfixed for 1 hour at 4ºC in darkness in 1% OsO_4, 1% $K_3Fe[CN]_6$ in water. After three rinses in distilled water at 4ºC, ovaries were stained for 2 hours at room temperature (RT) in darkness in 0.5% uranyl acetate, rinsed again in distilled water and dehydrated through an ethanol rising series (50%, 70%, 90% and 3x100%; 10 min. each) at RT and infiltrated with Embed 812 resin (Electron Microscopy Sciences). The resin-embedded specimens were polymerized by incubation in fresh Embed 812 during 48 hours at 60 ^{0}C in flat plastic embedding molds, which were cut in 50-70 nm thick sections with a DIATOME diamond-blade fixed on a Reichert Jung Ultramicrotome and mounted on copper grids. Sections were counterstained with 1% uranyl acetate in 50% ethanol for 1 min. and then stained with lead citrate for 5 minutes in a CO_2-free atmosphere. Sections were examined with a Zeiss EM902 electron microscope at 80Kv, and photographed at 50.000x magnification.

Atomic Force Microscopy measurements
Ovarioles were dissected out of the muscle sheath to make sure that the AFM cantilever was in direct contact with the basement membrane. Stiffness of *ex-vivo* ovarian tissues immobilized on Petri dishes using Cell-Tak (BD Biosciences, Oxford, UK) and containing *Drosophila* culture media was tested by AFM within 30 minutes of dissection. Monodisperse polystyrene beads (diameter 5.46 ± 0.12 μm, microParticles GmbH, Berlin, Germany) were glued to silicon cantilevers with a nominal spring constant of 0.1 N/m (PPP-BSI, Nanosensors, Neuchatel, Switzerland). Cantilevers were mounted on a JPK Nanowizard Cellhesion 200 (JPK Instruments AG, Berlin, Germany) set up on an inverted optical microscope. Exact cantilever spring constants were determined using the thermal noise method included in the AFM software. Freshly dissected samples were measured under visual control (Franze et al., 2011). Force-distance-curves were taken at an approach speed of 10 μm/s and a maximum force F = 6 nN. Force–distance curves were analyzed for an indentation depth d = 0.2 μm using a custom algorithm based in Matlab (Christ et al., 2010) (MathWorks, Natick, USA), which fits the Hertz model to the data: $F = \frac{4}{3} K \sqrt{R} \delta^{3/2}$, where the apparent elastic modulus $K = \frac{E}{1-\nu^2}$ is a measure of elastic stiffness, R is the radius of the indenter, E is the Young's modulus, and n is the Poisson's ratio. Normal distribution of AFM measurements was confirmed using the Kolmogorov-Smirnov test. The statistical significance of the differences between experimental and control values was evaluated using two-tailed t-tests. 58 measurements from 11 control ovarioles and 48 from 10 *laminin* mutants were collected.

Quantification of migration speed and fluorescent levels
The tracking of individual cells was performed using Fasciclin3::GFP (Fas3::GFP), Histone2Av::RFP (His2Av:mRFP) or transmitted light (Movies S1-S11). Lineal velocity of follicle cells was calculated by manually tracking nuclei or geometrical cell centres using the Leica LAS AF software, which allowed precise determination of time and length of movement. Dispersion of the migration speed data was represented using box-plot graphs, where the median and the outliers are indicated.

To quantify fluorescence in control and experimental samples and to avoid unwanted variability, both genotypes were dissected in parallel and fixed and immunostained in the same tube. To distinguish between experimental and control samples in a given experiment, we expressed a fluorescent marker (either nuclear GFP or His2Av::mRFP) in only one of the genotypes. In addition, quantification was performed on images captured using identical confocal settings. Z-sections were taken at 0.8-1.0 μm intervals. Colour depth was set to 12-bit and configured so that most of the pixels were within the dynamic range of the detectors. Z-Depth was selected manually and FITC, GFP or Cyanine5 fluorescence intensity signals were quantified in the plane of maximum

signal of the BM (laminin and Col IV) or of the basal surface of the follicle cells (integrin). Analysis was only done on consecutive egg chambers of ovarioles outside of the muscle sheath and from at least three biological replicas. Fluorescent intensity was measured in five different areas per 3+ egg chambers (Fig. S5). Image stacks were converted to grey scale and pre-processed using the standard background subtraction function of ImageJ (default settings; 50 pixel radius). Measurements were taken using IMARIS software with the "Measurement points" tool and/or the "ROI measurement" tool from the ImageJ software choosing the Z plane with maximal fluorescence signal. The "ROI measurement" tool allowed us to calculate the mean grey values for each area (Fig S5). Mean grey value equals the sum of the grey values of all the pixels in the selected area divided by the number of pixels and it is expressed in arbitrary units (A.U.). Egg chambers were staged according to (Spradling, 1993). Basically, we used the shape of the chamber, the morphology of the follicular epithelium and stalk cells, chromatin organisation in the nurse cells and the oocyte/nurse cells size ratio to assign the different stages.

Quantification of basal actin bundle and Col IV fibril alignment

One-plane confocal images were acquired and rotated to orientate the AP axis of the ovariole so that anterior is to the left. The orientation of both types of filaments was calculated by determining the angle between each filament and the AP axis of the ovariole (in the germarium and S1 follicles), or the axis defined by the polar cells (from S2-S8), using the line ROI tool of FIJI (Schindelin et al., 2012). Angular data was distributed from -90° to 90°, being 0° a perpendicular alignment. Angles were grouped in 10° intervals, and the percentage of filaments included within each interval was plotted as radial diagrams. Actin bundle alignment was determined from the germarium to S8 follicles; Col IV (*vkg::GFP*) fibrils were quantified in S5-S8 egg chambers.

SUPPLEMENTAL REFERENCES

Christ, A.F., Franze, K., Gautier, H., Moshayedi, P., Fawcett, J., Franklin, R.J., Karadottir, R.T., and Guck, J. (2010). Mechanical difference between white and gray matter in the rat cerebellum measured by scanning force microscopy. Journal of biomechanics 43, 2986-2992.

Franze, K., Francke, M., Günter, K., Christ, A.F., Körber, N., Reichenbach, A., and Guck, J. (2011). Spatial mapping of the mechanical properties of the living retina using scanning force microscopy. Soft Matter 7, 3147.

Kumagai, C., Kadowaki, T., and Kitagawa, Y. (1997). Disulfide-bonding between Drosophila laminin β and γ chains is essential for α chain to form αβγ trimer. FEBS Lett 412, 211-216.

Schindelin, J., Arganda-Carreras, I., Frise, E., Kaynig, V., Longair, M., Pietzsch, T., Preibisch, S., Rueden, C., Saalfeld, S., Schmid, B., et al. (2012). Fiji: an open-source platform for biological-image analysis. Nat Meth 9, 676-682.

Spradling, A. (1993). Developmental genetics of oogenesis. In The Development of Drosophila melanogaster, M. Bate, and A. Martinez-Arias, eds. (New York: Cold Spring Harbor Laboratory press), pp. 1-70.
